# Nanotechnology in Cancer Therapy: How Nanoparticles Are Shaping the Future of Personalized Treatment

**DOI:** 10.1021/acsnanomed.5c00117

**Published:** 2026-03-06

**Authors:** Indrianita Lionadi, Amir Farokh Payam

**Affiliations:** Nanotechnology and Integrated Bioengineering Centre, School of Engineering, 67075 Ulster University, Belfast BT15 1AP, United Kingdom

**Keywords:** cancer nanomedicine, targeted drug delivery, nanoparticle-based therapeutics, clinical translation challenges, combination therapies

## Abstract

Nanotechnology has transformed cancer research, with nanoparticles emerging as powerful platforms for therapy and diagnosis due to their tunable physicochemical properties and biocompatibility. Nanoparticles enable targeted drug delivery, improved pharmacokinetics, and reduced systemic toxicity compared with conventional therapies while also supporting molecular imaging and theragnostic applications. More than eight nanoparticle-based formulations are currently FDA/EMA approved for oncology, highlighting the clinical relevance of nanomedicine. Despite these advances, clinical translation remains limited by biological and technical challenges, including variability in the enhanced permeability and retention effect, insufficient tumor penetration, and low manufacturing scalability. This review primarily focuses on preclinical and translational nanomedicine with selected clinical examples. We summarize recent progress in major nanocarrier classes, including liposomes and polymeric, inorganic, and hybrid nanoparticles, and their roles in cancer therapy. These findings underscore that effective nanotherapeutic strategies must be informed by tumor biology rather than relying on passive targeting alone, that combination and stimuli-responsive platforms represent the most promising therapeutic avenues, and that meaningful clinical translation will depend on overcoming tumor heterogeneity with patient-specific treatment options and achieving robust, reproducible manufacturing.

## Graphical abstract

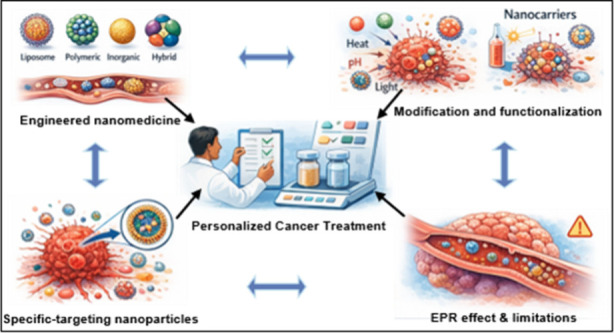

## 1 Introduction

The emergence of nanotechnology has catalyzed significant advancements across a wide array of scientific and technological disciplines, laying the groundwork for transformative innovations that continue to impact human health and well-being. At the heart of this revolution are nanoparticles (NPs), fundamental building blocks of the nanoscale world. These tiny structures operate at the interface of disciplines, enabling applications that span from medicine to materials science.^1^

In the field of medicine, nanoparticles have become pivotal due to their precision, biocompatibility, and functional versatility.^2^ They play a crucial role in targeted drug delivery, enabling therapeutic agents to reach specific cells or tissues with remarkable accuracy, thereby minimizing side effects and enhancing therapeutic outcomes.^1^ Moreover, their capacity to carry contrast agents or fluorescent dyes has opened new frontiers in molecular imaging, facilitating early disease detection and improved diagnostics.^3^ The excitement surrounding nanoparticles stems from their tunable physicochemical properties, high surface-to-volume ratio, and ability to be surface-functionalized, making them ideal for the development of novel biomaterials, biosensors, and nanodevices in medical and biological contexts.^4^

In healthcare technologies, nanoparticle applications can be broadly divided into two major sectors: (i) the design of advanced biomaterials,^5^ and (ii) the development of biosensors, diagnostic platforms, and drug delivery systems.^6,7^ Despite valid public concerns and ongoing investigations into nanotoxicity mechanisms,^8^ research in this area remains highly active and promising.

Cancer represents a major and escalating global health burden that has persisted for decades. It is one of the leading causes of mortality worldwide, accounting for nearly 10 million deaths annually—approximately one in six deaths globally—while also exerting profound socioeconomic consequences.^9^ The worldwide prevalence of cancer is projected to exceed 40 million cases by 2040, driven by population aging, environmental exposures, and lifestyle factors.^10^ In parallel with its clinical impact, cancer imposes an extraordinary economic burden, with cumulative global costs estimated at $25.2 trillion (in constant 2017 international dollars) between 2020 and 2050, corresponding to an effective annual loss of 0.55% of global gross domestic product.^9^ These converging trends underscore an urgent need for therapeutic strategies that improve the efficacy, reduce toxicity, and optimize resource utilization. Precision medicine has emerged as a central paradigm in oncology, aiming to tailor diagnosis and treatment based on individual tumor biology, genetic heterogeneity, and patient-specific factors. However, the clinical translation of precision oncology remains constrained by pharmacokinetic limitations, off-target toxicity, and inadequate tumor accumulation by using conventional delivery approaches. These challenges have catalyzed a growing interest in nanotechnology as a powerful platform to operationalize personalization in cancer care.

Nanomedicine offers distinct advantages derived from precise control over size, surface chemistry, and functionalization, enabling engineered nanoparticles to modulate biodistribution, enhance tumor targeting, and improve the therapeutic index. Clinically validated nanocarriers, including liposomal- and polymer-based systems, have demonstrated the ability to improve drug solubility, prolong circulation, and reduce systemic toxicity. Recent advances extend beyond passive targeting to include ligand-mediated active targeting, stimuli-responsive drug release, and multifunctional nanoplatforms that integrate therapeutic and diagnostic capabilities. Importantly, nanotechnology enables the codelivery of multiple agents, facilitating combination therapies tailored to tumor-specific molecular profiles and resistance mechanisms.

This review focuses specifically on nanoparticle-based strategies for cancer therapy. Although the concept of using nanoparticles as drug carriers is not limited to oncology, their role is particularly critical in this domain. Traditional drug administration, such as oral delivery, results in systemic exposure—meaning the dosage is distributed across the entire blood volume (∼4.5 L in adults). This often leads to inefficient targeting, high dosages, and severe side effects.^11^ In contrast, nanodrug carriers offer the potential to home in on specific organs or even individual cancer cells dramatically reducing required dosages and improving treatment specificity.

This targeted approach is especially relevant in the context of cancer, where chemotherapy and radiotherapy, although effective, often cause debilitating side effects. The integration of nanoparticles into both therapeutics and diagnostics offers a promising path forward, enabling earlier detection and more effective personalized interventions.

Over the past two decades, a number of specific reviews have already been published in this research area.^12–15^ The current work focuses on the most recent advances in drug delivery and specific targeting methods for cancer therapy with the use of nanoparticles. This review is structured around three core elements: Cancer Characteristics, Cancer Therapy, and Nanoparticles in Cancer Therapy, as illustrated in Figure 1. These elements are explored across five key sections: the biological features of cancer cells, current therapeutic approaches, functional roles of nanoparticles in cancer treatment, advancements in nanoparticle-based therapeutic strategies, and a comprehensive overview of targeting methods using nanoparticles. The last two sections will discuss the clinical translation status of established cancer nanomedicines and the outlook of personalized cancer treatment alternatives. While not exhaustive, this review aims to present a clear overview of the current state of the art and highlight recent advances in the field.

**Figure 1:**
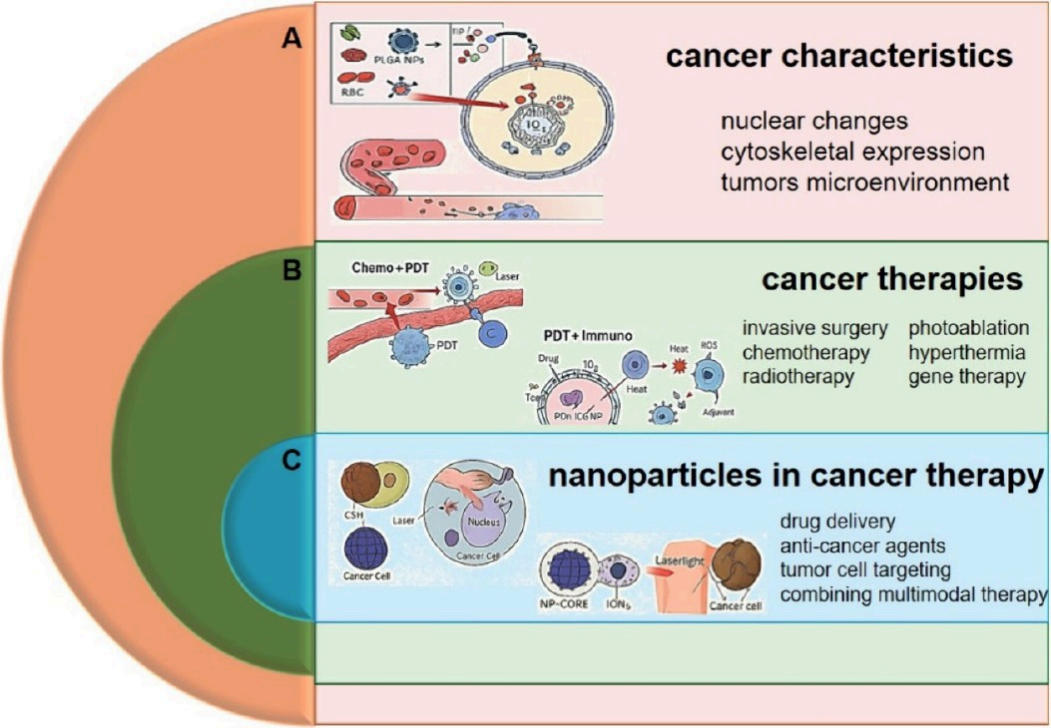
Outline of topics discussed in this review. This schematic illustrates the overarching structure of the review, organized into three main thematic components: (A) The biological and pathological characteristics of cancer, including cellular heterogeneity, uncontrolled proliferation, and mechanisms of metastasis; the representative images emphasize the nuclear changes of cancerous cells and their cytoskeletal expression with a proposed hybrid nanoparticles of PLGA-RBC (polylactic-*co*-glycolid acid with red blood cells) to penetrate intracellularly toward the cancer cells. (B) Current cancer therapy modalities, encompassing conventional approaches such as chemotherapy, radiotherapy, immunotherapy, and emerging targeted treatments; some examples of multimodal therapy include chemotherapy+PDT­(photodynamic therapy) and PDT+immunotherapy are depicted by the related images. Lastly, (C) focuses on the application of nanoparticles in cancer therapy, highlighting their multifunctional roles in drug delivery, diagnostics, imaging, and therapeutic targeting; the incorporated illustrations represent engineered nanoparticles as therapeutic agents such as core–shell nanoparticles (CSN) and core nanoparticle (NP-CORE) functionalized with ions. These nanoparticles are able to deliver anticancer drugs and simultaneously act as photothermal agents under laser exposure, thus enhance the therapy efficacy.

## 2 Cellular and Microenvironmental Features of Cancer

In this section, we briefly discuss the development of cancerous cells and their characteristics that lead to the development of cancer therapy approaches. Cancer cells result from genetic mutations that activate proto-oncogenes and deactivate tumor suppressor genes from a normal cell.^16^ These mutations lead to cellular changes, including insensitivity to growth signals, uncontrolled replication, apoptosis evasion, angiogenesis, and metastasis. Cancer cells also evade the immune system and alter metabolism, contributing to genetic instability.^17^ Metastasis is the spread of cancer cells beyond their original boundaries to invade other types of tissues and disseminate throughout the body, contributing to the lethality of the cancer cells. With all these changes, one can learn and recognized normal from cancer cells by observing their cellular properties and tumor-specific biomarkers surrounding cancerous cells.^18^ In general, there are three areas of characterization to recognize and differentiate cancer cells (Figure 2): the irregularities of nuclear structure, cytoskeletal rearrangements, and the tumor microenvironment (TME). The idea of optimizing cancer treatment is based on the fact that by learning the distinctive features of the cancerous cells, more specifically tailored treatment methods can be developed to effectively targeting cancer cells with high specificity. This section is covering the recent discoveries about the distinctive properties between normal and cancer cells that can possibly lead to specific targeting of cancers.

**Figure 2:**
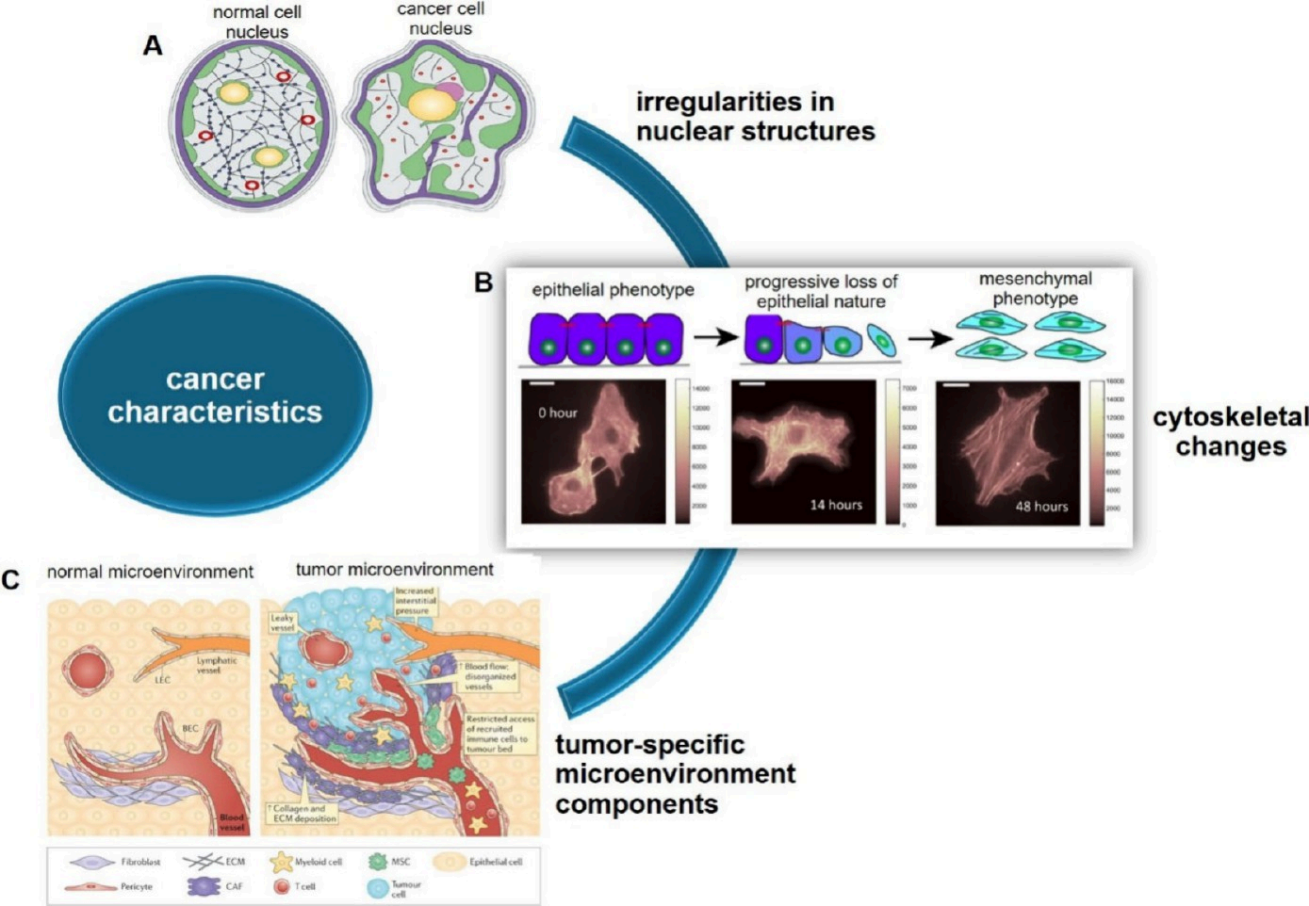
Overview of key structural and microenvironmental characteristics of cancer cells. This composite figure highlights three hallmarks of malignant transformation: (A) Nuclear alterations, including enlarged and irregularly shaped nuclei, which reflect chromatin remodeling and genomic instability (Reproduced with permission from ref 19. Copyright 2004 Springer Nature). (B) Cytoskeletal rearrangements, such as disrupted actin filament organization and altered cell polarity, contributing to enhanced motility and invasiveness (Reproduced from ref 20. Available under a CC-BY license. Copyright 2022 Arkaprabha Basu et al./Rearranged from original). (C) Tumor-specific tumor microenvironment (TME) features, including dense extracellular matrix (ECM), stromal remodeling, and immune cell infiltration, which support tumor progression and therapeutic resistance (Reproduced from ref 21. Copyright 2016 American Chemical Society).

### 2.1 Nuclear Architecture and Transformation

In normal cells, the nucleus is enclosed by the nuclear lamina, a proteinaceous layer composed of Lamins and associated proteins that anchors chromatin to the inner nuclear envelope and maintains nuclear mechanics and genome organization.^22^ In cancer, tumor microenvironment heterogeneity—including mechanical stress, hypoxia, and extracellular matrix remodeling—frequently alters lamin expression and nuclear architecture, increasing nuclear deformability and influencing intracellular nanoparticle trafficking and nuclear access.

Chromatin organization further reflects this heterogeneity. While chromatin in normal cells is predominantly heterochromatic, cancer cells often exhibit chromatin decompaction and spatial reorganization, which can modify nuclear permeability and affect the retention and efficacy of nanocarrier-delivered therapeutics. Nucleoli, typically present as 1–3 per nucleus and responsible for ribosome biogenesis, also regulate cellular proliferation and p53 metabolism.^19^ Enlarged and increased numbers of nucleoli in aggressive tumors may therefore contribute to heterogeneous nanoparticle–nucleus interactions and variable therapeutic responses.

The nuclear matrix provides a nonchromatin scaffold that organizes chromatin loops and nuclear substructures, introducing an additional layer of intracellular heterogeneity relevant to nanoparticle localization, particularly for nucleic acid–based delivery systems. Among these substructures, promyelocytic leukemia (PML) bodies play a central role in apoptotic regulation.^23^ PML bodies form multiprotein complexes within the nucleus (Figure 3), and their dysregulation in cancer may modulate nanoparticle-induced stress and apoptosis pathways.

**Figure 3:**
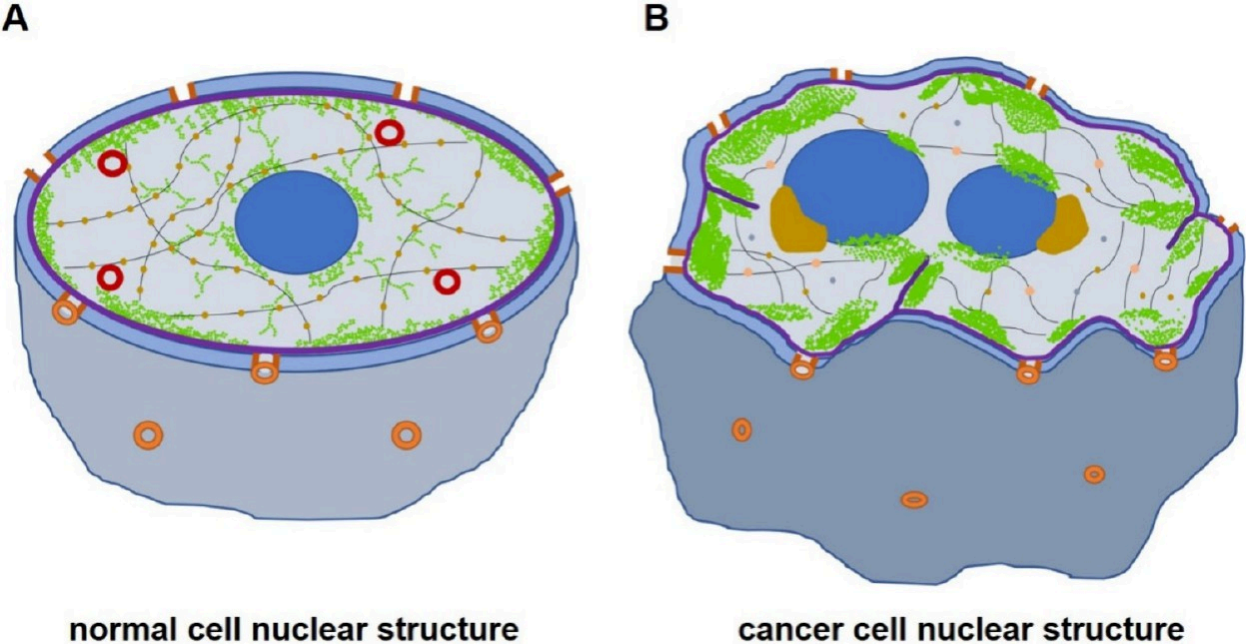
The physio-morphological differences between the nuclear anatomy of normal cell (left) and cancerous cell (right). In normal cells, the nuclear membrane is relatively smoother and spherical. There are evenly spread clusters of chromatins (green), a structurally ordered nuclear matrix (black lines), and the corresponding protein (orange) binding, typically with one spherical nucleolus and PML bodies (red) that contain tumor suppressor agents. In cancerous cells, the nuclear membrane is rough and irregularly shaped due to the coarse clustered chromatin and folded nuclear lamina (purple). There are also more than one nonspherical nucleoli observed with a perinucleolar compartment (dark yellow) that plays significant roles in cancerous progressions. The nuclear matrix is also found to be shorter and binded with anomalous proteins that are specific to the cancer type.

In cancerous cells, significant alterations in the nuclear structure occur, particularly involving the nuclear matrix, nuclear membrane, and chromatin. These structural changes are dynamic and vary according to both the type of cancer and its stage. Nuclear matrix proteins exhibit stage- and cancer-specific expression patterns, with certain proteins being confined to the nuclear matrix of specific cancer cells. The reorganization of the nuclear matrix in cancerous cells can lead to changes in DNA architecture, which subsequently affect processes such as gene expression, DNA replication, and other essential nuclear functions.^24^ Therefore, the composition of proteins within the cancerous nucleus is altered in a manner that is closely linked to the modifications occurring within these cells.

These nuclear alterations in cancerous cells such as disrupted chromatin organization, changes in nuclear matrix composition, and irregular nucleolar activity not only mark key hallmarks of malignancy but also present unique opportunities for targeted intervention. In this context, recent advances in nanotechnology have facilitated the use of functionalized nanoparticles to exploit these nuclear changes. Nanoparticles can be engineered to selectively localize within the tumor microenvironment and interact with cancer-specific nuclear components, including aberrant chromatin and nuclear matrix proteins.^25,26^ This enables their use as diagnostic tools, therapeutic delivery vehicles, or imaging contrast agents, offering new possibilities for precise and early detection as well as personalized treatment of cancer.

The morphological distinctions between normal and cancerous cells are most evident in the alterations of their nuclear structure, which include changes in the nuclear size, shape, nucleolar count, and chromatin distribution (see Figure 3). Table 1 summarizes the distinctive nuclear features between normal and cancerous cells.

**Table 1:** Comparison of Nuclear Features in Normal and Cancerous Cells

Feature	Normal Cells	Cancerous Cells
***Nuclear Lamina***	Composed of Lamins and associated proteins; Supports the nuclear envelope and Chromatin organization	Altered structure; Part of the broader nuclear structural changes are seen
***Nuclear Envelope***	Double membrane structure attached to nuclear lamina	Shows structural abnormalities depending on cancer type and stage
***Chromatin***	Predominantly heterochromatin; Well-organized; Contacts nuclear lamina and nucleoli	Disorganized; Changes in the architecture affect gene expression and DNA replication
***Nucleoli***	1–3 per nucleus; Involved in ribosome biogenesis, mRNA transport, Cell proliferation, and p53 metabolism	Often increased in number and size; Reflect elevated biosynthetic activity
***Nuclear Matrix***	Nonchromatin scaffold containing Nuclear Matrix Proteins (NMPs); Organizes chromatin and Subnuclear structures	Reorganized; NMPs show cancer- and stage-specific expression patterns
***PML Bodies***	Doughnut-shaped structures in the nuclear matrix; Regulate apoptosis in coordination with tumor suppressors	Structure and function often disrupted; Impact apoptosis regulation
***NMPs (Nuclear Matrix Proteins)***	Consistent and organized pattern of expression	Altered composition; Cancer-type and stage-specific profiles
***Nuclear Morphology***	Consistent size and shape; Defined chromatin distribution	Enlarged and irregular in size; Altered shape, Increased nucleolar count, and Disrupted chromatin pattern

Despite the attractiveness of cancer-specific nuclear alterations as intracellular targets, the effective engagement of these nuclear components by nanoparticles is ultimately constrained by the heterogeneous tumor microenvironment (TME) that governs nanoparticle transport and retention at the tissue scale. Tumor vasculature exhibits high variability in vessel density, permeability, and perfusion—factors that underlie the substantial inter- and intratumoral heterogeneity of the enhanced permeability and retention (EPR) effect in human cancers and contribute to low and inconsistent nanoparticle accumulation across tumor types and patients in clinical observations and meta-analyses. Studies report that clinical nanomedicines often achieve only a small fraction of the injected dose in solid tumors, with evidence of highly variable EPR-mediated delivery across different human tumors, challenging the assumption that passive accumulation alone ensures effective tumor exposure.^27–29^ Beyond vascular variability, dense and spatially heterogeneous extracellular matrix (ECM) networks elevate interstitial fluid pressure and sterically hinder diffusion, resulting in perivascular confinement and reduced penetration depth, while immune infiltration and stromal cell populations can sequester or clear nanoparticles before they reach tumor cells.^30^ Consequently, even nanoparticles engineered to recognize aberrant chromatin or nuclear matrix proteins may access nuclear targets only in subsets of cells that reside in TME regions permissive to transport and retention. This multiscale heterogeneity underscores that nuclear abnormalities represent necessary but not sufficient conditions for effective nanoparticle-based targeting in solid tumors and highlights the need to integrate intracellular target specificity with strategies that address TME-dependent transport barriers, which will be discussed more in later sections.

### 2.2 Molecular Alterations in the Nucleus

Cancerous cells exhibit several nuclear abnormalities including irregular nuclear margins, nuclear grooving, and convolutions. Nuclear shape changes in cancer cells lead to alterations in chromatin organization and gene positioning.^31,32^ These alterations are influenced by changes in Lamins, interactions between proteins like retinoblastoma (RB) and the inner nuclear membrane (INM), and mutations in the RET/PTC proto-oncogene. The nuclear membrane, responsible for regulating transport through nuclear pores, is often disrupted in cancer cells, impairing the transport of nuclear content and mislocalizing proteins, which, in turn, affects chromatin organization. At the INM, proteins such as LAP2α and lamin A/C interact with histones to regulate gene silencing.^33^ These interactions, alongside those with RB, initiate chromatin modifications and lead to epigenetic changes in gene expression, further contributing to cancer progression. The nuclear matrix, a critical 3D scaffold that supports nuclear structure and facilitates DNA organization, RNA synthesis, and gene regulation,^34,35^ also undergoes significant alterations. Changes in NMPs have been linked to cancer progression and are being explored as potential biomarkers for early stage cancer detection and metastasis assessment,^36^ with MAR­(matrix attachment region)-binding proteins are also showing promise as cancer therapy targets.^37^ For instance, the nucleophosmin (B23) protein is uniquely present in the nuclear matrix of prostate cancer cells,^38^ NMP179 is found in cervical cancer cells,^39^ and NMP22 (NUMA) is detected in the nuclear matrix of bladder cancer cells.^40^ These cancer-specific NMPs are increasingly being explored as potential biomarkers for clinical diagnosis. Table 2 comprises various NMPs reported in the literature, together with their specificity, clinical approval status, and corresponding diagnostic/prognostic information. The table highlights room for improvements in the NMP studies in conjunction with nanoparticles for cancer detection and targeting.

**Table 2:** Nuclear-Matrix Proteins Found as Biomarkers of Specific Cancer Cells

Biomarker	Organ Site (Cancer Type)	Approval Status	Diagnostic/Prognostic Insight	Nanoparticle Targeting	ref
*NMP22*	Bladder	√ FDA-approved (BladderChek)	Sensitivity 56–97%, Specificity ∼28–88%	No direct NP targeting, but sensor-based NMP22 biosensors developed	^41^,^42^
*BLCA-4 (NMP variant)*	Bladder	× Experimental	Promising early stage detection, ∼65–69% sensitivity and 88% specificity in limited cohorts	Not targeted by NPs	^43^
*Mcm5 + NMP22 combo*	Bladder	× Research use only	Improved diagnostic yield of Urothelial cell carcinoma diagnosis and identifies 95% of clinically significant disease	No NP targeting reported	^44^
*NM23 (NDPK)*	Various (Breast, Colon, Neuroblastoma)	× Prognostic marker only	Variable: inversely correlated with metastasis in some cancers	Rare NP gene delivery studies (preclinical)	^45^
*Osteopontin-c (NMP-related nuclear variant)*	Breast	× Experimental	High specificity for metastatic potential (∼75% detected in breast cancer), more highly suitable disease progression marker as it is not subject to the background noise resulting from the physiologic production of Osteopontin in the breast (it is absent from healthy cells)	Not NP-targeted	^46^
*NRP/B*	Brain (oxidative stress)	× Research	The failure of NRP/B mutants to activate Nrf2-mediated NQO1 may be associated with human brain tumor development	Not targeted by NPs	^47^
*PHB (Prohibitin)*	Breast	× Experimental	PHB participates in and stabilizes an important epigenetic chaperone HIRA complex in breast cancer cells. Accordingly, overexpression of nuclear PHB upregulates the HIRA complex-controlled H3.3 enrichment and increases the level of mesenchymal markers and finally induces the EMT in breast cancer	Not yet NP-targeted	^48^
*14-3-3β (phosphoserine/phosphothreonine), aldehyde dehydrogenase 1 (ALDH1)*	Colorectal	× Prognostic signature (IHC confirmed)	showed as the most prognostically significant and providing evidence for further dysregulation of protein expression in metastasis	Not NP-targeted directly	^49^
*catenins α-1, β-1, and δ-1, FUBP1 (or FUSE-binding protein 1), YBX1*	Colorectal	× Experimental	Differentially enriched in colorectal cancer tissues	No NP reports yet	^50^
*B23*	Prostate Cancer Cell Lines (LNCaP, DU-145, PPC-1, PC-3, and TSU)	× Research/Preclinical	Highly overexpressed in prostate cancer nuclei vs normal tissue shown by strong IHC staining	No NP reports yet	^38^
*NPM1 (nucleophosmin)*	Prostate Adenocarcinoma (LNCap)	× Preclinical Prognostic	Knockdown reduces cell migration/invasion, clonogenic growth, and tumor size in xenografts	No NP reports yet	^51^
*Purine-Rich Element Binding Protein α (PUR-α)*	Prostate	× Emerging Research	identified as a nuclear matrix protein implicated in prostatic cancer progression	No NP reports yet	^52^
*PARP and SATB1 (MAR-binding NMPs)*	Prostate Carcinoma Cells	× Proteomic research	Changes in these proteins composition across LNCaP, 22Rv1, PC3 cell lines indicate tumor progression	No NP reports yet	^53^
*Heterogeneous nuclear ribonucleoprotein K (hnRNP K)*	Prostate Carcinoma Cells	× Proteomic research	hnRNP K is significantly overexpressed in PCa with respect to NT tissue; moreover, an acidic isoform of this protein is exclusively present in tumor cells	No NP reports yet	^37^

In malignancies like small-cell lung carcinoma, the absence of Lamin A/C results in flexible nuclei that impact chromatin attachment points and heterochromatin formation.^54^ Additionally, thickened nuclear margins and peripheral heterochromatin are indicative of chromatin disorganization, contributing to altered gene expression and malignant transformation.^55^ These chromatin changes are also further influenced by chromosomal repositioning, histone acetylation modifications,^56^ and the altered expression of gene-rich and gene-poor chromosomes in cancer cells. Cellular senescence, a natural anticancer mechanism, can counteract this transformation by silencing proliferation-promoting genes through heterochromatin reorganization^57,58^ as the cell mechanism to suppress carcinogenic growth.

The nucleolus, which is responsible for rRNA synthesis, is also frequently altered in cancer cells. Nucleolar enlargement is commonly observed in tumors such as large-cell lung carcinoma, reflecting increased ribosomal biogenesis. However, this enlargement is also associated with alterations in p53 and pRB pathways, not solely with higher cell proliferation rates.^59–62^ The perinucleolar compartment (PNC), a meshwork found on the nucleolar surface, is rarely present in normal cells but is common in cancer cells, suggesting its potential as a diagnostic marker for cancer progression, particularly in the metastatic stage of breast cancer^63,64^ especially as it is accompanied by the loss of standard NMPs and the appearance of cancer-specific proteins. Furthermore, the promyelocytic leukemia body, which is misregulated in various cancers, plays a significant role in tumor progression. Loss of PML expression is correlated with increased invasiveness, highlighting PML as a potential therapeutic target in cancer treatment.^65,66^ Table 3 summarizes the general nuclear architecture changes in cancerous cells.

**Table 3:** Nuclear Architecture Alterations in Cancer Cells and Their Diagnostic Implications

Nuclear Feature	Abnormality in Cancer Cells	Mechanisms/Key Proteins	Functional Consequences	Clinical/Diagnostic Relevance
***Nuclear Shape***	Irregular margins, grooving, convolutions	Lamin alterations, RET/PTC mutations, RB-INM interaction	Altered chromatin organization and gene positioning	Indicator of malignancy
***Nuclear Membrane***	Disruption, impaired transport	Lamin A/C, LAP2α, altered INM protein interactions	Mislocalized nuclear proteins, disrupted chromatin structure	Loss of nuclear integrity in cancer
***Chromatin Organization***	Disorganized, peripheral heterochromatin	Lamin A/C loss, chromatin repositioning, histone acetylation, gene-rich/poor expression	Aberrant gene expression, malignant transformation	Target for epigenetic therapies
***Nuclear Matrix (NMPs)***	Altered protein composition, 3D disorganization	NMPs: B23 (prostate), NMP179 (cervical), NMP22 (bladder), MAR-binding proteins	Altered gene regulation, RNA synthesis, potential biomarker utility	Biomarkers for early diagnosis and metastasis
***Nucleolus***	Enlargement, functional abnormalities	Increased rRNA synthesis, p53/pRB pathway disruption	Reflects malignancy beyond proliferation	Diagnostic marker (e.g., large-cell lung carcinoma)
***Perinucleolar Compartment (PNC)***	Prominent in cancer, absent in normal cells	Linked to loss of standard NMPs, cancer-specific protein appearance	Associated with metastasis	Diagnostic marker, especially in breast cancer progression
***Promyelocytic Leukemia (PML) Bodies***	Downregulation or loss	PML misregulation	Increased invasiveness	Therapeutic target in tumor suppression
***Cellular Senescence***	May be bypassed or defective	Heterochromatin reorganization, gene silencing	Failed tumor suppression via proliferative arrest	Understanding mechanism offers new therapeutic targets

### 2.3 Cytoskeletal Remodeling in Cancer Cells

The cytoskeleton, a complex network of biopolymeric molecules, plays a pivotal role in determining cell shape and mechanical properties, including deformability and stiffness.^67^ These mechanical properties are crucial for key cellular processes such as mechano-transduction, proliferation, migration, and invasion.^67^ The composition and molecular architecture of the cytoskeleton, along with the surrounding chemomechanical environment, influence how a cell responds to mechanical forces and interacts with neighboring cells and the extracellular matrix (ECM). As a result, alterations in these mechanical properties can serve as indicators of a cell’s biological state, offering valuable insights into pathogenic mechanisms and distinguishing between normal and cancerous cells.^68^

In cancerous cells, the cytoskeleton is essential for maintaining cell integrity and supporting processes such as division, shape, migration, and invasion. The cytoskeletal network consists of actin microfilaments, intermediate filaments, and microtubules, all of which are regulated by the nucleus.^69^ Genetic mutations within the nucleus can lead to corresponding changes in the expression of cytoskeletal components, making these structural changes potential biomarkers for cancer detection. Furthermore, defects or alterations in the cytoskeleton’s mechanical properties such as increased rigidity or changes in cell deformability can drive cancerous behavior, including abnormal migration and invasion.^70^ These mechanical changes are closely linked to various types of tumors and other diseases. Targeting the cytoskeleton’s structure, mechanics, and biochemical functions offers a promising therapeutic approach for cancer treatment. Notably, studies have shown differential expression of keratins between normal and cancerous mammary epithelial cells,^71^ and the unique coexpression of cytokeratin and vimentin in metastatic cancer cells is linked to enhanced cell development and progression.^72^ Given their ability to interact with cytoskeletal elements and modulate mechanical or signaling pathways, nanoparticles have emerged as versatile tools for both probing cytoskeletal dynamics and delivering cytoskeleton-targeted therapies in cancer research.^73^

### 2.4 Tumor Microenvironment and ECM Changes

Alterations in nuclear arrangement within cancer cells trigger changes throughout the cell, leading to distinctive behaviors compared to normal cells. These behaviors are reflected in the types of molecules found in the ECM and their interactions with surrounding structures.^74^ Cancerous cells create a robust ECM barrier, aiding their defense against the immune system and supporting their mobility, which enables metastasis to other tissues.^74^ Identifying specific ECM components in cancer cells is crucial for developing targeted therapies.

The TME, where cancer cells reside, plays a central role in tumor initiation, progression, and therapeutic resistance.^75^ It is composed of immune cells, fibroblasts, endothelial cells, blood vessels, ECM, signaling molecules, and cancer stem cells.^75^ These components interact dynamically with tumor cells to support their growth, survival, and metastasis. Key features of the TME such as hypoxia, chronic inflammation, and immunosuppression contribute to immune evasion, drug resistance, and reduced infiltration of therapeutic agents.^75^ Innate and adaptive immune cells in the TME can shift toward tumor-promoting phenotypes, while intratumorally microbes have been shown to modulate immune responses and influence outcomes in immunotherapy. Due to its critical role in shaping cancer behavior and treatment outcomes, the TME is now a major focus in cancer research.^76^ Therapeutic strategies aim to reprogram the TME by alleviating immunosuppression, restoring immune cell function, and improving drug delivery for more effective treatment.^77^ Hence, nanoparticles offer a powerful platform to modulate the TME by enabling targeted delivery of immunomodulatory agents, improving drug penetration, and reshaping immune responses within the tumor site.^25^

Tumor vasculature, extracellular matrix (ECM) density, and immune infiltration collectively shape how effectively nanoparticles penetrate solid tumors. Abnormal tumor vasculature—characterized by irregular, leaky, and heterogeneous microvessels with poor perfusion and impaired lymphatic drainage—can facilitate enhanced permeability and retention (EPR)-mediated extravasation of nanoparticles, but this phenomenon is highly variable across human tumors and often insufficient to drive consistent clinical accumulation without adjunct modulation strategies. Clinical and preclinical evidence indicates that the EPR effect varies substantially between tumor types, between patients, and even within different regions of the same tumor, contributing to inconsistent nanomedicine delivery in humans relative to small-animal models.^28,78^

Once nanoparticles exit the vasculature, ECM density and composition act as major physical barriers: dense networks of collagen, hyaluronic acid, and proteoglycans increase interstitial fluid pressure and sterically hinder diffusion, confining nanoparticles near perivascular regions and limiting deep penetration into tumor tissue. ECM modulation (e.g., enzymatic degradation or stromal cell targeting) has been shown to enhance nanoparticle diffusion and improve therapeutic distribution.^79,80^

Immune infiltration further influences nanoparticle distribution: although infiltrating immune cells can transport or clear nanoparticles, immunosuppressive microenvironments and physical barriers associated with stromal ECM often restrict effective immune cell access, reducing opportunities for immune-mediated nanoparticle trafficking into deeper tumor regions. Nanoparticles engineering strategies that engage immune cells or modulate immune-ECM interactions are being explored to improve penetration and therapeutic outcomes.^80^

### 2.5 Diagnostic Markers and Targeting Strategies

Cancer progression is typically classified into three stages: benign, malignant, and metastatic. Benign tumors are localized and noninvasive, often resembling normal cells in structure but exhibiting abnormal growth behaviors.^81^ Malignant tumors breach tissue boundaries, invading surrounding tissues, whereas metastatic tumors possess enhanced adaptability and disseminate systemically.^82^ The treatment strategies required for each stage vary accordingly: while benign tumors may respond well to surgical or localized therapy, malignant and metastatic cancers demand more advanced targeting mechanisms to achieve specificity and minimize systemic toxicity.^82^

One of the principal methods for identifying cancer cells lies in observing nuclear and chromatin abnormalities, which result in altered mechanical properties, such as increased nuclear deformability, reduced stiffness, and changes in viscoelasticity.^19,83^ These mechanical changes are associated with heterochromatin remodelling and alterations in the intermediate filament network (e.g., keratin), which collectively contribute to abnormal cellular morphology and enhanced metastatic potential.^84^

Another critical factor is the tumor microenvironment which consists of a complex interplay between tumor cells and noncancerous components such as immune cells, fibroblasts, and extracellular matrix elements.^85,86^ The TME plays a pivotal role in tumor survival, immune evasion, angiogenesis, and therapeutic resistance. Certain TME characteristics, such as hypoxia, acidity, and abnormal vasculature, are conserved across many cancer types, providing shared biomarkers and therapeutic targets.

Cancer targeting strategies now employ this growing understanding of cancer biology to improve the selectivity and efficacy. Rather than relying solely on nonspecific treatments such as radiation or cytotoxic chemotherapy, modern approaches focus on targeting molecular signatures and biomechanical properties unique to cancer cells.^87^ These may include surface receptors, epigenetic profiles, or specific mechanical deformability.^88^ Advances in imaging and diagnostic techniques further enhance early detection and therapeutic precision.^89^ Table 4 summarizes biological markers derived from cancerous cells followed by their diagnostic/targeting relevance.

**Table 4:** Summary of Cancer Cell Features and Targeting Approaches

Feature	Cancer-Associated Changes	Diagnostic/Targeting Relevance	Ref
*Nuclear Structure*	Irregular margins, grooves, enlarged nucleoli	Imaging, nuclear biomarkers (e.g., NMPs, PML bodies)	^19^,^90^
*Chromatin Organization*	Heterochromatin loss, histone modifications, chromosomal repositioning	Epigenetic therapy, chromatin-targeting drugs	^83^,^84^,^88^
*Nuclear Membrane Proteins*	Lamin A/C loss, altered LAP2α, disrupted transport	Mechanobiological markers, structural drug targets	^19^,^90^
*Mechanical Properties*	Reduced stiffness, higher deformability	Deformability-based isolation and microfluidic sorting	^91–93^
*Nuclear Matrix Proteins (NMPs)*	Cancer-specific NMPs (e.g., B23, NMP22)	Cancer diagnostics, early stage detection	^37^,^38^,^94^
*Tumor Microenvironment (TME)*	Hypoxia, acidic pH, ECM remodeling, immune modulation	Immunotherapy, TME-targeted delivery	^26^,^76^,^95^,^96^
*Therapeutic Targeting Approaches*	Molecular targeting, biomechanical profiling, surface modification detection	Improved specificity, reduced toxicity	^97–99^

## 3 Established Cancer Treatment Modalities

Therapy methods aimed at treating various cancerous cells that have been currently applied range from removal surgery, photoablation, hyperthermia, chemotherapy, radiation therapy, and immunotherapy. Figure 4 and Table 5 summarize the cancer therapy methods.

**Figure 4:**
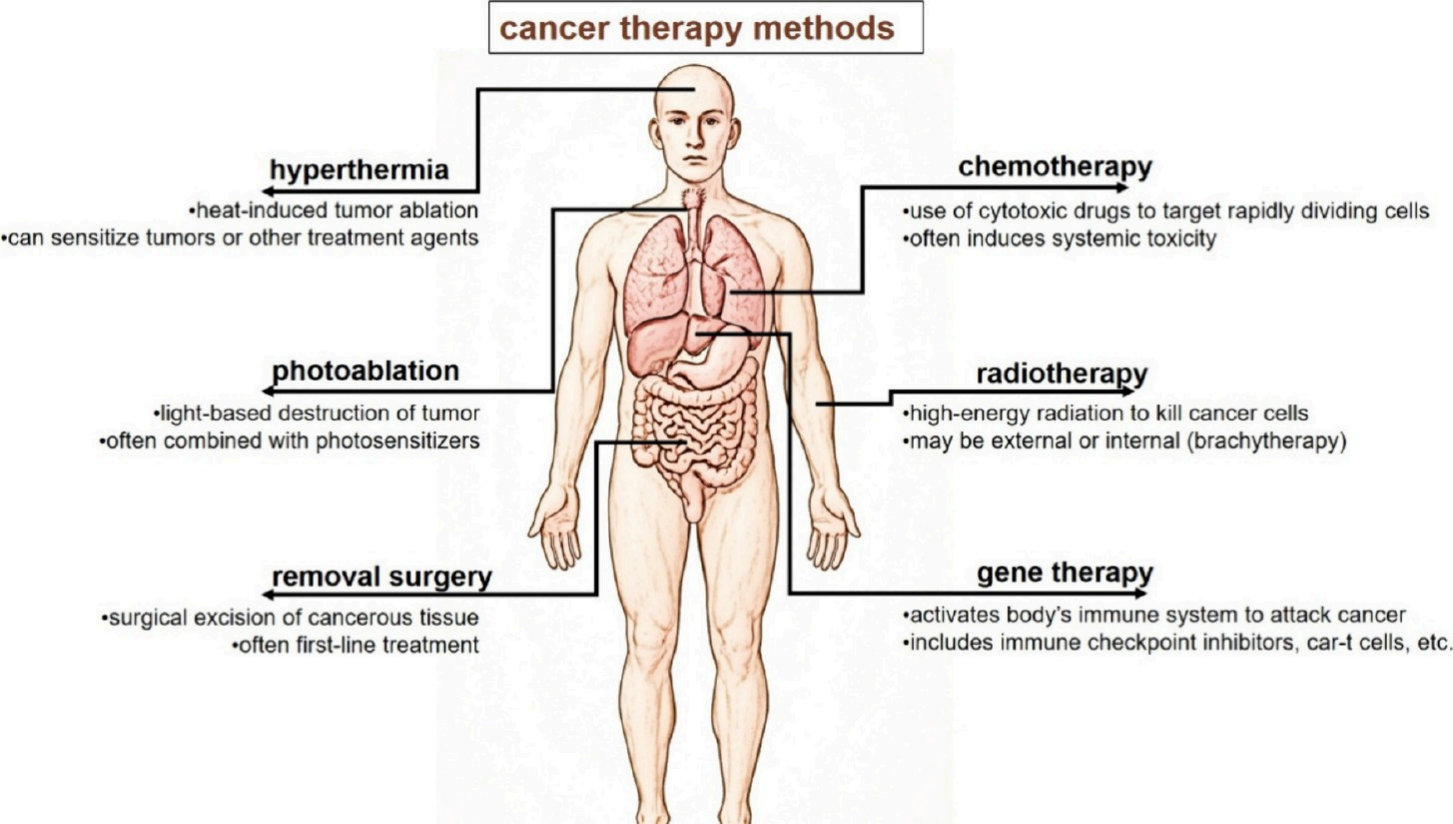
Established cancer therapy modalities and their primary targets. Schematic overview of major cancer treatment strategies currently used in clinical practice, including surgical resection, photoablation and hyperthermia, chemotherapy, radiation therapy, and immunotherapy. Each modality is illustrated with its principal anatomical and cellular target regions in the human body, highlighting differences in the treatment localization and systemic involvement. The figure provides a framework for comparing conventional therapies and for contextualizing how nanotechnology-based approaches can enhance targeting precision and therapeutic efficacy.

**Table 5:** Cancer Therapy Methods with Their Advantages, Limitations, Applications, and Roles of NPs within Each Modality

Therapy Method	Advantages	Limitations	Applications	Roles of NPs
*Removal Surgery*	Quick and straightforward	Includes the removal of some part of the body, which could lead to unnatural changes in the body and various side effects	Has been applied in most of the cases of cancer occurrence	NPs are used in some of the surgical tools to precisely locate the target cancer cells within the body
	Highly effective, supported by advanced procedure	High chances of cancer cells to grow back, both in the same or different part of the body		
		Limited to some local area of the body		
				
*Chemotherapy*	Effectively kills the cancerous cells and slows down their growth	Due to its toxicity, it affects other healthy cells within the body, arising serious side effects	Most of well-known cancer such as breast cancer, lung cancer, prostate cancer, colorectal cancer, and melanoma	NPs can be used as drug delivery agents to effectively target the cancer cells and hold drug release until it arrives at the target cells
	Can be used to reach the spreading cancer cells	Needs a very careful and precise dosing		
		Developing the multiple drugs resistance		
				
*Radiotherapy*	Destructs localized cancer cells without invasive treatment	Limited to some local part of the body, ineffective for a spreading cancer	Most of the less-spreading cancers and cancers near the surface of the body such as skin cancer	NPs are used as a radiosensitizer to enhance the efficiency of radiotherapy on the target cells (in brachytherapy)
		High energy beam will also arise unwanted side effect to the adjacent healthy cells		
				
*Photoablation*	Highly reduced effect on healthy cells due to the effective cancer targeting	Limited to a short distance from the skin surface since the light used cannot pass through more than 1 to 3 in. from the skin	Used to treat cancer around or underneath the skin such as esophageal cancer	NPs are the agent for photosensitizer that go toward the cancerous cells and releases reactive oxygen species (ROS) under light irradiation which able to destruct the cells containing them, or simply generate heat that leads to cell apoptosis
				
*Hyperthermia*	Able to eliminate the drug-resistant and radio-resistant cancer cells	Needs of highly precised and stable equipment	Has been put as a standard treatment for cervical cancer and several types of sarcomas	NPs can be aimed to reside on targeted cells to enhance thermal response and thus improve efficiency of the therapy
	Less invasive procedure	Difficult to determine exact temperature for specific cancer cells	Reported to be successful in treating brain, bladder, rectal and esophageal cancer	
	Less harmful for adjacent healthy cells	Induces metabolic changes of the healthy cells to some extent		
	Highly effective when applied in vivo	Inducing thermotolerance effect to the affected cells		
		Mostly still needs to be combined with other therapy methods to enhance effectivity		
				
*Gene Therapy*	Highly flexible and personalize-able according to the specific cancer case	Inefficient cellular uptake	A number of studies are still ongoing to bring it closer to clinical applications, in combination with other therapy methods	NPs are vital as the cell targeting agent with their passive and active targeting
	Effectively targets specific tumors and less likely to affect healthy cells	Instable in the body fluid	Most studies are done for pancreatic cancer, prostate cancer, bladder cancer, breast cancer, lung cancer and other malignant melanomas	NPs allows combination of therapy methods for cancer treatment since they have multiple beneficial properties that can be used to address several therapy methods with a proper functionalization
	Much reduced side effects	More resources need to be allocated to ensure the effectiveness in clinical practice		
	Acts as a vaccine that reduces metastasis and prevents cancer reoccurrences			

### 3.1 Removal Surgery

Cancer removal surgery is a procedure to surgically remove the organ or tissue part where the cancerous cells are found.^100,101^ It is still the first-line treatment for most of the cancer cases where a much-increased advancement in precision and invasiveness minimalization are possible.^100,101^ Variation of monitoring techniques, and artificial intelligence are enhanced to assist current cancer removal procedures.^102^ However, this may not be applicable for all cases, as it strongly depends on physiological conditions and effective location of the tumors. Removal operations are said to be possible if the cancers are localized, have not yet developed complex angiogenesis, and have not interacted with main blood vessels.

Nanoparticles are transforming cancer surgery by improving intraoperative precision and tumor visualization.^103^ One major application is tumor localization, where quantum dots (QDs) conjugated with antibodies have enabled precise imaging of tumors in vivo. Gao et al.^104^ demonstrated this in murine models using both active targeting and passive enhanced permeability and retention (EPR), showing that QDs can help make tumors visible to surgeons or imaging systems.

A critical surgical challenge is achieving clear tumor margins especially in breast cancer, where up to 55% of patients need reoperation due to residual disease.^105^ Nanoparticles like CLIO-Cy5.5­(cross-linked iron oxide with fluorescence dye), a dual MRI and near-infrared fluorescent agent, have been used to delineate tumor margins more clearly. Weissleder et al.^106^ showed that it accumulates in tumors and guides resection through optical imaging, improving margin control.

Nanoparticles also aid in identifying vital adjacent structures, such as nerves, vessels, and ureters, to avoid surgical injury. Near-infrared dyes like indocyanine green have been used to visualize ureters during surgery in animal models, reducing the risk of injury.^107^

For sentinel lymph node mapping, nanoparticles provide real-time lymphatic tracking. Frangioni et al.^103,108^ used QDs in pig models were used to successfully identify and resect sentinel nodes in melanoma. Their fluorescence also guided precise histological analysis, showing potential for broader surgical application.^103^

Finally, detecting residual microscopic tumor cells is crucial for complete resection. Many tumors leave behind subclinical deposits undetectable by the human eye, which can lead to recurrence.^109^ Nanoparticles like QDs and SERS agents enhance contrast and signal intensity, improving detection of such cells during surgery and potentially increasing cure rates.^110^

### 3.2 Chemotherapy

Chemotherapy is the introduction of anticancer drug to the body to prevent or even stop the cancer cells multiplication.^111^ The anticancer drugs used are designed to interfere with rapidly dividing cancerous cells and deactivate them. The main challenge in chemotherapy treatment is the nonbeneficial side effect, that is the damage of the untargeted healthy cells.^112^ The clinical use of chemotherapy has been improved according to the case of the dosing regimens and combination with other treatments with adjuvant or neoadjuvant administration. Nanoparticle applications on chemotherapy have been widely studied to improve its effectiveness by improving the targeting delivery.^113^ Yücel et al.^114^ reported their experiment on modifying spherical AuNPs with glutathione-coated folic acid and loaded it with Methotrexate, a widely used anticancer drug. The results show that this modification not only enhanced the cytotoxicity of methotrexate but also showed an effective targeted drug delivery.

By enabling targeted and sustained drug delivery, nanoparticles can minimize damage to healthy tissues while maximizing therapeutic efficacy.^115^ Various nanoparticle types—such as liposomes, dendrimers, and quantum dots—offer unique physicochemical properties (size, shape, surface charge) that influence their biological interactions and treatment outcomes.^116^

Both passive targeting (via the enhanced permeability and retention effect) and active targeting (through surface functionalization with ligands or antibodies) have enhanced precision in drug delivery.^117^ Advances in nanoparticle design have also enabled the codelivery of multiple drugs, as well as stimuli-responsive systems that release drugs in response to pH, temperature, or enzymatic triggers.^118^

In vitro and in vivo studies have demonstrated the efficacy and safety of these nanotherapeutics, with some outperforming standard chemotherapy in reducing tumor burden and systemic toxicity. However, challenges remain in clinical translation, including immune clearance, scale-up, and regulatory hurdles. Nonetheless, ongoing innovation in material science is driving the development of next-generation nanoparticles with improved targeting and biocompatibility.^119^

### 3.3 Radiotherapy

Radiation therapy, also called radiotherapy (RT), uses high energy beams to destroy cancer cells. Radiation of high energy beams can disturb the genetic material inside the cancer cells and disable the cells from multiplication, leading to cell death.^120^ Advancements have been implemented in clinical use of radiotherapy, including improved precision targeting and reduced destructive effects to the adjacent normal cells which are then more known as brachytherapy. Zhao et al.^121^ has reported the success of specific targeting for C6 glioma cells with AgNPs that have been functionalized with polyethylene glycol and aptamer As1411 to enhance the radio-sensitizing effect. It not only infiltrated into the tumor cells but also went deep into the core of the tumor spheroids and thus improved the overall efficacy of the radiotherapy method.

Radiotherapy remains a fundamental treatment modality for cancer, with nearly 50% of patients receiving RT during their course of treatment.^122^ RT targets tumors using ionizing radiation to kill cancer cells and has been applied to both solid and metastatic tumors. However, conventional RT is limited by off-target damage to healthy tissues, lower radiation intensity at distant tumor sites, and radiation resistance (RR)—a major contributor to treatment failure and tumor relapse.^123^

To overcome these limitations, nanoparticle-assisted RT has emerged as a promising strategy. Nanoparticles, especially those composed of high atomic number (Z) elements such as gold (Au), hafnium (Hf), or gadolinium (Gd), act as radiosensitizers by enhancing radiation absorption and increasing local dose deposition in tumor tissues.^124,125^ When irradiated, these NPs emit photoelectrons and Auger electrons that cause localized DNA damage and enhance tumor cell killing.^126^ Furthermore, the combination of NPs with RT induces reactive oxygen species (ROS), interferes with DNA repair pathways, and arrests the cancer cell cycle, thereby enhancing radiosensitivity.^127^

Importantly, NP-based RT has been shown to reduce damage to surrounding healthy tissues by limiting the bystander effect and improving tumor specificity. In addition, NPs can modulate the tumor microenvironment by alleviating hypoxia and reducing metastasis, improving overall treatment efficacy.^128^ When combined with phototherapy, nano-RT platforms can further minimize phototoxicity while providing complementary tumoricidal effects.^128^

### 3.4 Photoablation

There are two principal modalities of photoablation therapy distinguished by the nature of the therapeutic agents employed. Photothermal therapy (PTT) uses photothermal agents (PTAs) that absorb light and convert it into heat, inducing localized hyperthermia that damages cancer cells.^129^ In contrast, photodynamic therapy (PDT) involves light-sensitive molecules known as photosensitizers that are activated under specific light wavelengths. Upon activation, these agents generate electron–hole pairs, which react with water molecules or hydroxyl ions to produce reactive oxygen species (ROS)—cytotoxic agents that cause targeted cell death.^130^

A major challenge in both PTT and PDT is the efficient intracellular delivery of these agents to tumor cells. To address this, Qin et al. developed a multifunctional nanocarrier by coupling graphene oxide with Fe_3_O_4_ nanoparticles and chitosan to load photosensitizers for the treatment of hepatoma cells.^131^ Their in vitro studies demonstrated that the modified nanoparticles effectively penetrated the tumor nucleus and induced apoptotic cell death under light irradiation.

In a systematic review,^132^ numerous advancements in nanomaterial-based PTT for cancer treatment were examined in both in vitro and in vivo contexts. NPs have gained considerable attention due to their ease of synthesis, tunable surface functionalization, and physicochemical diversity making them ideal candidates for targeted PTT.^132^ Compared with conventional PTT, targeted-PTT, which involves directing NPs specifically to tumor tissues, significantly enhances therapeutic efficacy while minimizing damage to surrounding healthy cells.

Studies have extensively documented the anticancer potential of nanoparticle-assisted PTT, particularly under laboratory (in vitro) conditions. However, most of these studies involve diverse NP systems with limited evaluation of systemic biocompatibility or long-term safety, highlighting the need for further research.^133^ For clinical translation of targeted PTT, several key considerations must be addressed: (i) expanded in vivo studies to confirm the biocompatibility of used NPs, Thorough investigation of off-target, (ii) effects and long-term toxicity, (iii) customization of nanomaterials for tissue-specific applications, as a single nanomaterial may not be universally effective across cancer types.^134^

In addition to direct cytotoxicity, PTT can trigger immunogenic cell death, stimulating an antitumor immune response. This involves the activation of effector immune cells, secretion of cytokines, and differentiation of naïve T-cells into antigen-specific memory T-cells, potentially inhibiting tumor recurrence and supporting long-term tumor control.^135^

### 3.5 Hyperthermia

Thermal ablation has emerged as a preferred alternative to surgical resection for eliminating tumor cells. Thermoablation employs heat (>50 °C) to induce cancer cell apoptosis via protein coagulation.^136^ A milder form, known as hyperthermia (HT), operates at 41–48 °C and enhances treatment efficacy without directly killing cells.^137^ HT can improve tumor perfusion and oxygenation and sensitize hypoxic tumor cores to chemotherapy and radiotherapy. Even at 42 °C, prolonged exposure (e.g., 60 min) can induce necrosis.^137^

Despite its potential, the clinical use of HT is limited by challenges in temperature control and targeting. This has led to growing interest in nanoparticle-mediated HT (NP-HT), particularly using magnetic nanoparticles (MNPs) activated by an alternating magnetic field (AMF).^138^ NP-HT enables localized heating, image-guided delivery, and thermal monitoring, enhancing precision and minimizing off-target damage.^138^

MNP-based hyperthermia has shown promise in preclinical models and in early clinical trials. In one study, MNPs injected into prostate tumors successfully localized under imaging, and AMF exposure led to effective tumor ablation with minimal side effects.^139^

Key factors affecting MNP heating efficiency include particle size, shape, polymer coating, and AMF parameters.^140^ MNP-HT has also been shown to synergize with chemotherapy, radiotherapy, immunotherapy, and gene therapy, enhancing the overall anticancer efficacy. Furthermore, MNPs can serve as vehicles for targeted drug delivery, improving treatment specificity, and reducing systemic toxicity.

With ongoing advances in nanoparticle engineering and clinical validation, NP-mediated HT represents a promising integrative approach to cancer therapy, combining localized thermal ablation with systemic treatment enhancements.

### 3.6 Gene Therapy

Cancer is fundamentally a genetic disease driven by mutations in key genes that promote uncontrolled cell growth. Gene therapy offers a promising strategy to correct or modulate these mutations by delivering therapeutic genetic material into cancer cells, leading to reprogramming, growth inhibition, or apoptosis. However, challenges such as poor cellular uptake and instability in biological fluids continue to limit its therapeutic efficacy.^141^

Nanotechnology has significantly advanced gene therapy by improving targeted delivery, stability, and controlled release of nucleic acids. Surface-modified nanoparticles can be functionalized with targeting ligands to enhance the interaction with cell surface receptors. Optimizing parameters such as ligand density, hydrophobicity, and avidity have shown to improve biodistribution, cellular uptake, and therapeutic outcomes while reducing off-target toxicity.^142^

Among emerging gene therapy applications, cancer immunotherapy has gained significant clinical attention due to its ability to stimulate the patient’s immune system to recognize and destroy cancer cells. Unlike conventional treatments, immunotherapy also limits metastasis and recurrence. Strategies include immune checkpoint blockade, T-cell engineering, cancer vaccines, and delivery of immunomodulators.^143–145^ For example, Xu et al.^143^ combined immunotherapy with photothermal approach to improve immunotherapeutic efficacy. They occupied nanoengager (SPNE) which consisted of photothermal core coated with preengineered tumor cell membranes and dendritic cells as the cancer vaccine. The results demonstrate effective tumor growth inhibition and metastasis prevention on 4T1 tumor cells. Cancer immunotherapy methods include modification of T cells, increasing the immunological tolerance, immune checkpoint blockage, and identifying the novel tumor antigens with the use of next-generation sequencing. In the passive cancer immunotherapy, there are executive agents involved, such as monoclonal antibodies, cytokines, and lymphocytes.^144^ They can promote a resistant antitumor response. While in active cancer immunotherapy, active provocation to the self-immune response is created by vaccination, immunomodulation, or specific antigen receptor. Antitumor immunity can also be boosted by engineering the tumor immune environment, immunomodulators delivery, creating the subpopulation of tumor-specific immune cells, triggering innate immunity, prompting the immunogenic damage to the cancerous cells, and facilitating the infiltration of antitumor immune cells and their interaction with the target cancer cells.^145^

To address the limitations of gene delivery, stimuli-responsive nanocarriers are being developed that respond to environmental cues (e.g., pH, ROS, and enzymes) to release genetic cargo specifically in the tumor microenvironment. These “smart” systems take advantage of the enhanced permeability and retention (EPR) effect in tumors, allowing for selective accumulation and reduced systemic toxicity. New approaches like CRISPR/Cas9-loaded nanoparticles offer spatiotemporal genome editing, expanding precision therapeutic capabilities.^141,146^

Another promising avenue is the codelivery strategy, where multiple therapeutic agents (e.g., CRISPR, chemotherapy, or immunomodulators) are encapsulated in the same nanocarrier. This allows synergistic action, optimized dosing, and precise sequencing of the release. For instance, P-gp inhibitors must be released prior to chemotherapeutics to prevent premature drug efflux and maximize therapeutic effect.^146^

Macrophage-based nanocarriers represent a biologically inspired delivery system. Macrophages possess innate targeting, phagocytic capacity, and deep tissue penetration. They can carry nanoparticles loaded with drugs or genes, modulate the TME, and present antigens for immune activation. However, clinical translation faces challenges, including immunogenicity, phenotype stability, and scalability.^147^

Moreover, exosomes have emerged as natural nanocarriers for gene therapy agents such as siRNAs, ASOs, and antibodies. Their long circulation time, biocompatibility, and ability to cross the blood-brain barrier make them ideal candidates for gene delivery, particularly in neurological cancers. However, concerns about tumor-derived exosomes, large-scale production, and functional heterogeneity require further investigation.^148^

Overall, integrating nanotechnology with gene therapy and immunotherapy enables more precise, safer, and effective cancer treatments. Future efforts will focus on optimizing delivery platforms, improving clinical safety, and achieving scalable manufacturing to facilitate clinical translation. Table 5 summarizes the advantages and limitations of cancer therapy methods with the proposed role of nanoparticles to enhance the therapy efficacy.

## 4 Nanoparticles: Roles and Challenges in Cancer Therapy

With the advancement of various cancer treatment modalities, including previously discussed chemotherapy, radiotherapy, immunotherapy, and gene therapy, significant clinical challenges remain, particularly regarding minimizing off-target side effects and enhancing therapeutic efficacy. One of the central requirements across these modalities is the availability of stable, biocompatible carriers capable of transporting therapeutic agents directly to malignant tissues. These carriers should ideally not only protect the therapeutic cargo but also selectively accumulate in cancerous cells to facilitate localized action such as tumor suppression, apoptosis induction, or growth inhibition.

A key obstacle in conventional anticancer drug delivery lies in the low molecular weight of most therapeutic compounds, which results in nonspecific diffusion into healthy tissues and premature systemic clearance before they reach tumor sites.^115^ This lack of specificity contributes to systemic toxicity, multidrug resistance (MDR), and suboptimal treatment outcomes.^149^ Therefore, a targeted delivery strategy is essential one that enables rapid accumulation of the therapeutic agent at the tumor site while minimizing clearance by the reticuloendothelial system (RES) and evading biological barriers such as the blood-brain barrier and tumor stroma.^150^

Nanoparticles have emerged as a powerful solution to these challenges. Due to their tunable physicochemical properties including size, shape, surface charge, and ligand functionalization, NPs can be engineered to specifically target tumor tissues via passive mechanisms (e.g., the enhanced permeability and retention effect) or active targeting using surface ligands that bind to tumor-specific receptors.^151^ Once internalized, NPs can mediate subcellular trafficking, enhance endosomal escape, and even initiate specific biomolecular interactions required to induce therapeutic responses. Moreover, they can stimulate immune activation, facilitating synergistic action with immunotherapy agents.^152^

As a result of these multifaceted capabilities, NPs are at the forefront of cancer-targeted nanomedicine development. Their roles range from drug delivery and imaging enhancement to acting as therapeutic agents themselves through photothermal, photodynamic, or magnetic hyperthermia effects. An overview of the versatile applications of NPs across different treatment strategies is summarized in Table 5, highlighting how tailored nanoparticle platforms can enhance treatment specificity, efficacy, and safety in a broad range of oncological settings.

### 4.1 Beneficial Properties of Nanoparticles and the Following Challenges

NPs are widely recognized for their large surface area-to-volume ratio, tunable surface chemistry, and compositional versatility, making them highly suitable for crossing biological barriers and delivering therapeutic agents directly to cancerous tissues.^153^ In addition to their structural properties, NPs possess catalytic, magnetic, thermal, optical, mechanical, and electronic attributes that can be leveraged for multifunctional roles in cancer therapy. However, these inherent characteristics alone are not sufficient to achieve optimal therapeutic outcomes. Several limitations persist, including short circulation times, insufficient tumor specificity, and concerns about biological safety (Figure 5). Importantly, studies have demonstrated that the size, shape, and surface properties of NPs directly influence their toxicity, clearance, and accumulation in target tissues.^153–157^ For instance, silver nanoparticles (AgNPs), owing to their high surface area, can enter the human body through the skin, lungs, or bloodstream, where they increase reactive oxygen species (ROS), reduce adenosine triphosphate (ATP) levels, and impair mitochondrial function, leading to cytotoxic effects.^153^

**Figure 5:**
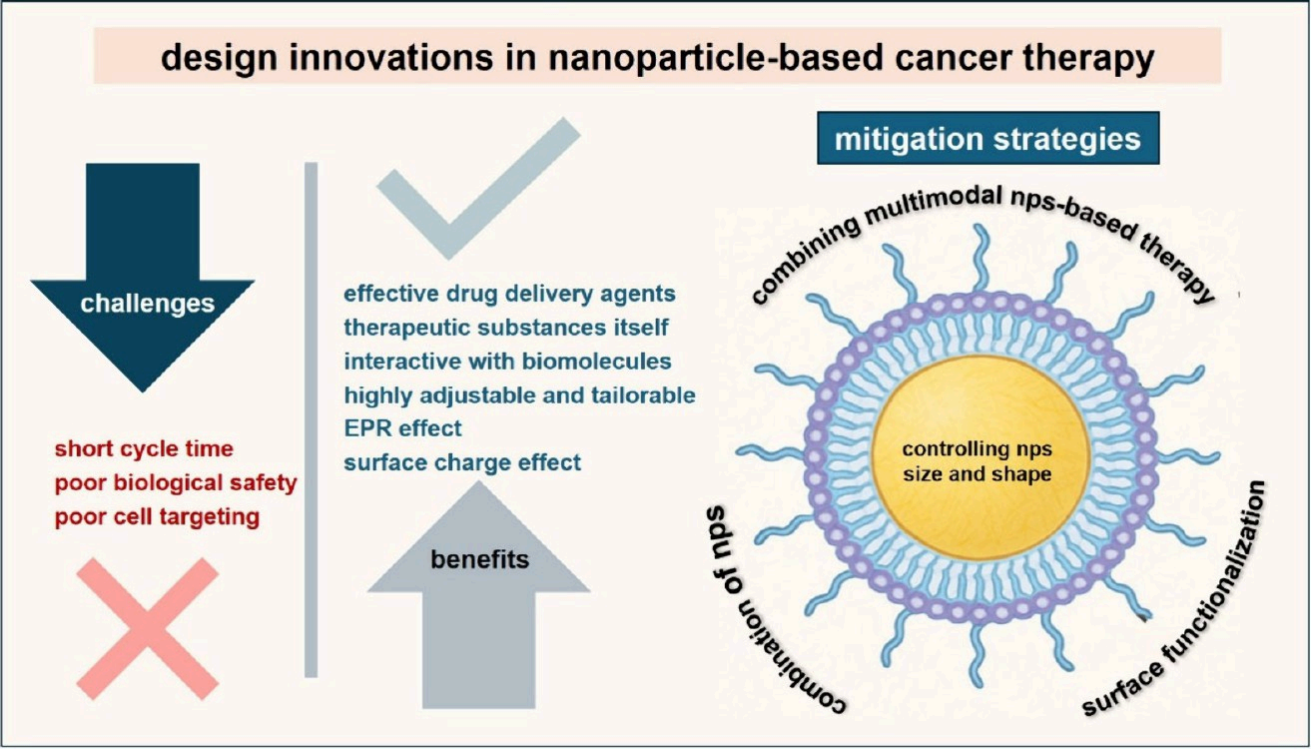
Visualized design innovations in nanoparticle-based cancer therapy depicting challenges and benefits of the application of NPs and the prospective developments as mitigation strategies to be done in NP-based cancer therapy, including 1. Combinations of various NPs as hybrid NPs; to combine photodynamic NPs with magnetic NPs to enhance localization, 2. Combining multimodal NPs-based therapy; to synergize cytotoxicity with thermal ablation, 3. Surface functionalization; biomarkers and specific protein coatings to enable active targeting and controlled release, and 4. Controlling the optimum size and shape of the NPs.

NPs utilize two main targeting approaches: passive and active targeting. In passive targeting, NPs exploit the enhanced permeability and retention effect (EPR), wherein the leaky vasculature and poor lymphatic drainage of tumors allow preferential NP accumulation. While EPR effect has been a foundational principle in nanomedicine design, accumulating clinical evidence indicates that the EPR effect is highly heterogeneous and often inconsistent in human cancers.^158^ Tumor vascular permeability, perfusion, and lymphatic dysfunction vary substantially not only between tumor types but also across patients and within different regions of the same tumor, resulting in uneven nanoparticle extravasation and retention.^28^ Meta-analyses and clinical imaging studies have demonstrated that, in contrast to preclinical models, many human solid tumors exhibit limited or spatially restricted EPR-mediated accumulation, with median nanoparticle delivery efficiencies frequently below 1% of the injected dose.^159,160^ Hypervascularized tumors such as sarcomas and colorectal carcinomas tend to display relatively higher EPR intensity, whereas hypovascularized and desmoplastic tumors, including pancreatic ductal adenocarcinoma and prostate cancer, show markedly reduced permeability and nanoparticle penetration.^28^ These observations underscore that EPR-driven delivery is context-dependent rather than universal and that reliance on passive targeting alone is insufficient to guarantee effective tumor accumulation (see Table 6). Consequently, rational nanomedicine design increasingly requires tumor-specific stratification and the integration of complementary strategies—such as active targeting, microenvironment modulation, or transport pathway engagement—to overcome EPR limitations in heterogeneous human tumors.

**Table 6:** Representative Variability of the EPR Intensity Across Human Tumor Types^a^

Tumor Type	Vascularization/Stromal Features	Relative EPR Intensity	Implications For NP Delivery
***Sarcoma***	Highly vascularized, heterogeneous vasculature	High	Favorable passive NP accumulation
***Colorectal Carcinoma***	Moderately to highly vascularized	Moderate–High	Variable but often effective EPR
***Breast Cancer***	Heterogeneous vasculature and ECM	Moderate	Strong interpatient variability
***Lung Cancer***	Variable perfusion and permeability	Moderate	Context-dependent NP delivery
***Pancreatic Ductal Adenocarcinoma***	Dense desmoplastic stroma, low perfusion	Low	Poor NP penetration, EPR-limited
***Prostate Cancer***	Hypovascularized, compact stroma	Low	Minimal passive accumulation

a Information summarized from refs 28 and 161 .

Active targeting involves surface functionalization of NPs with ligands, such as antibodies, peptides, or small molecules that bind selectively to receptors overexpressed on cancer cells. Hydrophilic polymers like poly­(ethylene glycol) (PEG) can enhance NP solubility, reduce immunogenicity, and prolong systemic circulation by avoiding mononuclear phagocyte system (MPS) clearance. This functionalization improves both tumor selectivity and cellular uptake, achieving cancer cell apoptosis with minimal toxicity to surrounding healthy tissues.^115^

In addition to size and ligand specificity, the surface charge plays a pivotal role in NP performance. Charge-reversal NPs—designed to be neutral or negatively charged under physiological conditions but switch to positive charge in the tumor microenvironment (e.g., under acidic pH or oxidative conditions)—can enhance circulation time while maximizing tumor uptake.^162,163^ For example, Wang et al.^162,163^ demonstrated that PEGylated, positively charged NPs penetrate tumor interstitial space more effectively than neutral or negatively charged counterparts. However, excessively high zeta potential can increase cytotoxicity and reduce colloidal stability, indicating that the surface charge must be finely tuned to maximize therapeutic efficacy while minimizing side effects.

Enhancing the EPR effect has also been a major focus of recent research. Strategies such as tumor ECM remodeling, vasculature normalization, or coadministration of agents like TNF-α, collagenase, or TGF-β inhibitors have shown promise in improving NP penetration and distribution within solid tumors.^77,145,164^

Multifunctional NPs that combine therapeutic and diagnostic roles—so-called theranostic agents—have also emerged. These can deliver chemotherapeutics, imaging agents, and even gene-editing tools simultaneously, offering a powerful platform for precision oncology.^165^

Ultimately, the NP size, surface charge, shape, and functionalization are key determinants of biodistribution, tumor penetration, and systemic toxicity. A deep understanding of the correlation between nanoparticle physicochemical properties and biological response is crucial for the rational design and clinical translation of nanomedicine.^162,163^ Figure 5 summarizes the principal benefits and remaining challenges in NP-mediated cancer therapy, followed by how design innovations can overcome those challenges.

### 4.2 Roles of NPs in Delivering Cancer Treatment

Nanomedicine has significantly improved cancer treatments, especially in immunotherapy application efficacy.^77,145,164,166,167^ Nanoemulsions, nanotubes, NPs and other engineered nanostructured materials offer a promising cancer immunotherapy efficacy. They can be nanocarriers that deliver immunologically functional and active elements to the appropriate targeted sites more effectively. Cancer-targeted nanomedicines with immunotherapy are aimed to boost anticancer immunity by modifying the cancer immune environment while delivering immunomodulators, creating cancer-specific immune cells initiator, enhance innate immunity, inducing immunogenic cell death to the cancer cells, and facilitate the infiltration and interaction of anticancer immune cells with the targeted cancer cells.

However, as discussed earlier, the TME itself is, in fact, the major factor that hinders cancer immunotherapy. It is well-known that TME has a detrimental effect on the immune cells that infiltrate the tumor cells. This damaging effect is due to its immunosuppressive features. It has become a remarkable barrier to deliver cancer immunotherapy, but NPs can adjust the TME to allow the enhancement of the cancer immunotherapy delivery.^168^ NPs play an important role in modulating the TME and augmenting the immunotherapy process.

There are various immunotherapy strategies that have been developed in cancer treatment scope, including ligand functionalized NPs, liposomal nano formulations, metallic NPs especially Au- and AgNPs, antigen-specific T cells, polystyrene NPs, iron-oxide NPs, etc. In the immunotherapy approach, the specific targeting delivery is followed by intracellular delivery of tumor-specific antigens or adjuvants to antigen-presenting cells (APCs) which can also be facilitated by NPs which thereby will activate the immune system. These APCs include B cells, T cells, dendritic cells (DC), and macrophages. In this way, NPs are utilized to employ, activate, and circulate the internal endogenic dendritic cells to produce dendritic cell-based vaccines to fight against cancer cells.^144,169^

## 5 Design Innovations in Nanoparticle-Based Cancer Therapy

So far, we know that NPs are useful in cancer treatment mainly because they can act as 1. An agent, or nanocarrier of the anticancer drug,^115^ 2. The photoablation agents,^170^ both in PTT or even better in PDT, or it is also possible for them to act as both at the same time. But as an agent or nanocarrier, NPs need to be able to overcome the barrier challenges to arrive at the target cells; these include the body immune system, the disturbance from other cells or tissue within the body, as well as the protection system of the targeted cell itself. Thus, the main idea of the effectivization of NPs application in cancer therapy is to direct the NPs movements in the body. The key approach to accomplishing this idea is, but not limited to, functionalizing the surface of the NPs. This process basically covers the NPs with substances that will modulate the interaction between the NPs and the surrounding substances or molecules. The combination of specific interactions that can allow the NPs to move along and act according to the way that they are designed can be tailored with surface functionalization. Another practical approach to the effectivization in using NPs in general is combining several treatment methods as a unified multimodal approach. NPs can serve as versatile platforms that enable the combination of multiple therapeutic modalities, including chemotherapy, photothermal therapy, photodynamic therapy, gene silencing/editing, immunotherapy, and radiation therapy.

Despite the substantial therapeutic promise of NPs in cancer therapy, their clinical translation remains constrained by nanotoxicity, which encompasses organ-level accumulation, mechanistic cellular toxicity, and immune system interactions (Table 7). A growing corpus of meta-analyses and systematic reviews demonstrates that the biodistribution of NPs following systemic administration is nonuniform and heavily influenced by physicochemical properties, such as size, surface charge, and coating. On average, NP accumulation is highest in the liver (median ∼10–11% injected dose), followed by the spleen and kidneys, with lower but still measurable levels in the lungs and heart.^171^ This pattern was consistently observed across studies in tumor-bearing animal models and indicates a dominant role of the reticuloendothelial system (RES) in NP sequestration.^171^

**Table 7:** Toxicological Concerns in Nanoparticle Cancer Therapeutics and Standard Assessment Assays

Toxicological Domain	Principal Organs/Systems	Mechanistic Insights	Standard Assays/Metrics	Ref
***Organ Accumulation***	Liver, spleen, kidneys, lungs, heart	RES uptake, macrophage sequestration; influences therapeutic index	*In vivo* biodistribution (radiolabel, fluorescence, ICP-MS), %ID metrics across organs	^171^
***Oxidative Stress* *and ROS***	All tissues, especially liver	ROS generation via redox surface reactions, mitochondrial disruption; lipid peroxidation, DNA damage	DCFH-DA ROS assays, antioxidant enzyme levels, lipid peroxidation assays	^173^
***Genotoxicity***	Nucleus/genomic DNA	DNA strand breaks, chromosomal alterations	Comet assay, micronucleus assay	^176^
***Immune Activation* *and Inflammation***	Blood, RES organs	PRR activation, inflammasome assembly (e.g., NLRP3), cytokine release	Complement activation assays (CH50, SC5b-9), cytokine panels (ELISA)	^174^
***Hemocompatibility***	Blood circulatory system	Hemolysis, platelet activation, coagulation disturbance	Hemolysis assay, platelet aggregation, coagulation time	^177^
***Organ Function Alterations***	Liver and kidney	Hepatocyte dysfunction, nephrotoxicity, biochemical changes	Serum ALT/AST, creatinine/BUN, histopathology/grading	^178^,^179^
***Chronic Retention/Clearance***	Reticuloendothelial System (RES) organs	Long-term retention and slow clearance	Longitudinal *in vivo* tracking, excretion profiling	^180^

Liver and spleen uptake is largely mediated by Kupffer cells and splenic macrophages, which act as primary clearance mechanisms but also represent major sites of off-target toxicity. Hepatic accumulation, in particular, is associated with elevated reactive oxygen species (ROS) production and pro-inflammatory cytokine release (e.g., TNF-α, IL-1β), which promote oxidative stress, hepatocyte damage, and potential fibrotic responses.^172^ Within cells, NPs can amplify ROS generation through surface redox activity and interactions with mitochondrial respiration pathways, leading to lipid peroxidation, DNA damage, and apoptosis or necrosis.^173^

In parallel, NPs can activate innate and adaptive immune pathways. Excess ROS and damage-associated molecular patterns (DAMPs) trigger pattern recognition receptors (PRRs) such as Toll-like and NOD-like receptors, leading to inflammasome assembly (e.g., NLRP3) and pro-inflammatory cytokine production. While controlled immune engagement may enhance antitumor responses, uncontrolled activation increases immunotoxic risk, including systemic inflammation or hypersensitivity.^174^ The interplay of ROS-mediated cytotoxicity and immune activation highlights a central nanotoxicity axis relevant to both therapeutic effects and adverse outcomes.

To systematically assess NP safety, researchers employ a suite of standard toxicological assays. *In vitro* genotoxicity tests (comet and micronucleus) evaluate DNA integrity, while hemocompatibility assays measure effects on red blood cells (hemolysis), platelet function, and coagulation pathways. Complement activation assays (e.g., CH50, SC5b-9) gauge the potential for infusion-related immune reactions, and *in vivo* biodistribution studies (using radiolabels, fluorescent tags, or mass spectrometry) quantify organ accumulation and clearance dynamics. Histopathological examination, serum biochemistry, and immune profiling further characterize organ-specific toxicity, especially hepatotoxicity and nephrotoxicity.

Importantly, nanotoxicity is not an immutable property of all NPs but is highly dependent on the design parameters and surface engineering. Meta-analyses reveal that surface functionalizations such as PEGylation can reduce macrophage uptake by 60–75% and extend circulation half-life, thereby improving safety profiles, while biodegradable coatings mitigate long-term retention in RES organs.^175^ Rational design that integrates toxicology early in development facilitates both therapeutic performance and regulatory progression.

Other than focusing on the tumor specific targeting and delivering to avoid nanotoxicity, additional advantages of NPs are also worth discussing. These are metastasis inhibition,^181^ multidrug resistance (MDR) reversal,^182^ and molecular targeting therapeutic.^183^ In term of metastasis inhibition, functionalized nanoparticles can inhibit epithelial-mesenchymal transition (EMT), a key process in tumor invasion and metastasis and may activate the host immune system to attack both primary and metastatic tumors.^184^ Another major advantage is their potential to capture and eliminate circulating tumor cells (CTCs), thereby preventing the formation of micrometastases an essential but challenging step in halting cancer spread and recurrence. On the other hand, chemotherapy failures are known to be linked to drug resistance, which allows tumors to survive and grow despite therapy. This often begins with a single agent and expanding to multiple drugs with similar mechanisms, a phenomenon known as multidrug resistance (MDR).^182^ Key mechanisms of MDR include suppressed apoptosis, enhanced DNA repair, drug inactivation, efflux protein overexpression, and miRNA involvement, as the cost of repeated introduction of drugs with poor targeting.^185,186^ Nanotechnology has emerged as a promising strategy to overcome MDR, offering improved tumor targeting, better pharmacokinetics, reduced toxicity, and enhanced therapeutic outcomes through both passive and active delivery systems.^185^ Approaches include nanoparticle-mediated siRNA delivery, drug combinations, and antibody targeting.^185^

Many nanomedicines are advancing toward clinical trials, although challenges remain, such as particle aggregation, limited drug loading, and burst release. This development leads to molecular targeting therapeutics, which are principally more efficient due to their specific intervention toward the progression, growth, and spread of the cancerous cells, while the classic anticancer drugs are prone to be prematurely deactivated and thus lost their function to kill the cancer cells. Molecular targeted therapies combat cancer by inhibiting cell proliferation, metastasis, and angiogenesis, inducing apoptosis, reversing drug resistance, and enhancing antitumor immunity.^187^ Some agents promote CD8+ T-cell and NK cell activity or trigger immunogenic cell death, especially in combination with chemotherapy.^188^ Despite their precision, molecular targeted therapies can still lead to side effects such as fatigue, skin rash, cardiotoxicity, and gastrointestinal symptoms.^187^ Resistance and relapse also remain significant hurdles. To address these, advances in genome sequencing, AI, and machine learning are being used to design better drugs, predict responses, and reduce toxicity.^189^ Additionally, gut microbiome is increasingly recognized as a factor in treatment response and side effects, though its role in targeted therapy specifically is still under investigation.^190^ Further research may enable microbiome-informed therapeutic strategies for improved safety and efficacy.

### 5.1 Surface Functionalization for Targeted Delivery

As discussed earlier, there are two ways of cell-targeting that can be applied to NPs; passive^191^ and active^117^ targeting. In short, passive targeting allows for the efficient localization of nanoparticles within the tumor microenvironment, while active targeting facilitates the active uptake of nanoparticles by the tumor cells themselves. Compared to normal tissues, tumors are mostly more solid due to their impaired lymphatic drainage, defective vasculature, and lower interstitial fluid.^192^ This disparity can be used for the selective accumulation of NPs in tumors, aided by the prolonged blood circulation feature that comes from surface modification which provides a stealth profile to the NPs. This mechanism is the EPR based passive targeting.^191^ Basically, accumulation of the tumor sites is possible as a consequence of the unique vasculature and poorly arranged lymphatic drainage system within the tumorous cells. However, improvements have been made to allow more active approaches to promote the antitumor immune cells, which are the active targeting of NPs.^193^ The functionalization of the NPs in this approach is not only to prolong their blood circulation extent, but also to make the NPs interact with the target cells by attaching specific ligands, including peptides, proteins, and antibodies that can match with the overexpressed receptors in the tumor site.^1^ Well-functionalized NPs are also able to regulate drug release through various external and internal stimuli, including ultrasound, light, radiation, pH, or glutathione, in order to avoid premature drug release within the healthy or untargeted cells. Figure 6 depicts the significance of active and passive targeting by the NPs.

**Figure 6:**
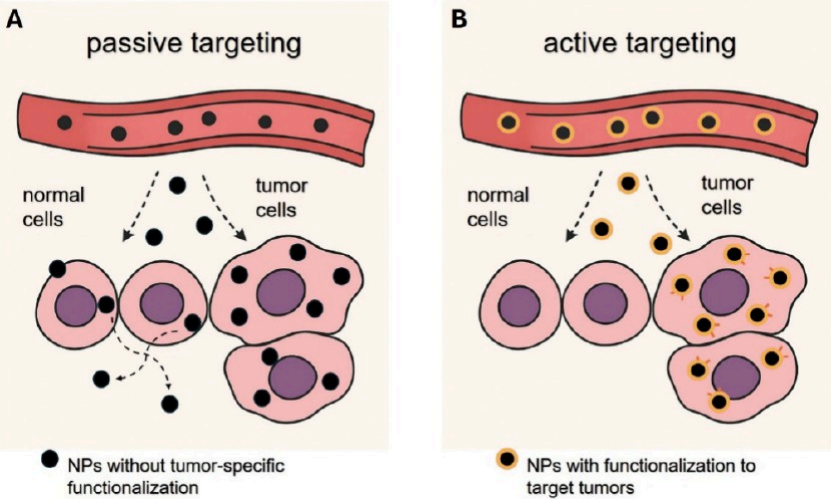
Schematic of different NPs targeting pathways. In passive targeting (A), NPs are accumulated more in tumor cells because they are ‘trapped’ by the poor vascularization system of tumorous cells, while they can also penetrate normal cells, they are not as accumulated because normal cells can ‘flush’ them out eventually. In active targeting (B), NPs are encapsulated by specific ligands or other moieties that can only interact with specific receptor within tumor cells, bypassing the normal cells, and thus improved effectivity is achieved.

There is a development of Cell membrane nanoparticles (CMNPs) that takes place to provide a way of optimizing nanomedicine efficacy. The main idea of CMNPs is to use the cell membranes of the target patients to achieve a personalized treatment while also using the NPs core that can load various drugs to adjust with the anticancer immune response. Cell membranes from the white blood cells and natural killer cells, as the examples, can be more beneficial as they would enhance immune factor secretion such as antitumor cytokines CXCL10 and interferon-γ.^194^ This approach will be discussed in more detail in the following section.

### 5.2 Biomimetic Nanoparticles for Targeted Therapy

Cell membranes are the main mediators where the exchange of information and signaling between living cells occur. Numerous recognition units, such as integral proteins and glycans, on the surface of cell membranes equip cells for a high level of biological specificity. The beneficial properties of the cell membranes, including targeting ability, immune escape and long blood circulation, can be bestowed on CMNPs to overcome challenges and limitations of NPs application in cancer therapy.^195^ Cancerous cells are known by the presence of specific plasma membrane proteins within their surface which enables them to proliferate massively, adhere to various isotypes, avoid clearance by the immune system effectively.^196^ There are also specific features within their membranes that allow them to recognize homologous cells and get close to each other, giving a self-localization function. Therefore, imparting these properties to the NPs through cancer cell membrane coating will improve the efficacy of therapeutic drug delivery to the target cancer cells. There are a number of studies conducted to develop the use of CMNPs in cancer treatment and gave a promising result.^197^ They combined the CMNPs with the common cancer cell therapy methods such as photoablation, immunotherapy, and chemotherapy.

As mentioned earlier in cancer therapy methods, photoablation is divided into two approach, PTT and PDT.^170^ In PTT method, a photothermal agent is irradiated by light with a certain wavelength in order to increase its temperature and injure the targeted cancer cells.^198^ In PDT method, photosensitizers are used instead to produce various ROS, which are detrimental to the target cancer cells, under a certain light irradiation.^199^ Both of these methods have one challenge in common; that is, to deliver the target drugs to the cancer cells without being removed by the immune system. This challenge can be overcome by developing CMNPs, which can transport the photothermal agents and the photosensitizers with or without additional drugs efficiently to the targeted sites. Chen et al.^200^ has successfully developed biomimetic NPs with indocyanine green (ICG)-loaded and coated with cancer cell membrane (ICNPs) which demonstrates significant cell endocytosis and increased homologous-targeting tumor accumulation *in vivo*. Their ICNPs were also reported to be good at disguising as cells and thus reducing interception by the excretion systems.

CMNPs have also been developed for their use in cancer gene therapy method. In cancer gene therapy, specific exogenous target genes are introduced into the targeted cancer cells using appropriate vectors to correct the overexpressed or faulty genes so that the cells can be reprogrammed to be normal cells.^201^ Again, the challenge of providing safe, yet effective, delivery of the target genes is still there to be solved, especially when it comes to clinical application. And thus, CMNPs can be utilized to help improve the delivery process of the genes by enhancing cancer site targeting, the circulation time of the drugs, and the gene transfection efficiency. A study has reported a positive result of using CMNPs in gene therapy method with in vitro experiment to a mouse model of lung metastasis of melanoma.^202^

As the most widely applied treatment for cancer therapy, chemotherapy has an obvious disadvantage; that it blindly attacks cancer cells together with the adjacent healthy cells, resulting in remarkable side effects that degrade the general health condition of the patients. Therefore, further development to improve chemotherapy drug delivery is necessary to diminish the damage of the healthy cells. Combination of CMNPs with chemotherapy drugs is one of the most recent developments for targeted chemotherapy drug delivery. Sun et al.^203^ developed a strategy to target the homogeneous cancer cells using the nanoparticle agents loaded with paclitaxel that were coated with cancer cell membranes. These prepared nanodrugs have the high targeting ability and antimetastasis effect for the specific corresponding cancer cells.

In a broader context, nanoparticle targeting strategies can be broadly categorized into passive, active, and stimuli-responsive mechanisms, as schematically illustrated in Figure 7. To summarize, passive targeting exploits tumor-specific pathophysiological features—such as leaky vasculature and impaired lymphatic drainage—to enable preferential nanoparticle accumulation via the enhanced permeability and retention (EPR) effect, particularly when prolonged circulation is achieved through surface modification. Active targeting further improves specificity by functionalizing nanoparticles with ligands that recognize overexpressed receptors on tumor cells, thereby promoting receptor-mediated endocytosis and enhanced intracellular delivery. Beyond these approaches, there is stimuli-responsive targeting that integrates internal cues (e.g., pH, redox gradients, enzymes) or external triggers (e.g., light, ultrasound, magnetic fields) to achieve spatially and temporally controlled drug release. Together, these complementary strategies provide a unified framework for understanding how rational nanoparticle design can overcome biological barriers and improve therapeutic precision, forming the conceptual basis for advanced platforms such as biomimetic and cell membrane–coated nanoparticles.

**Figure 7:**
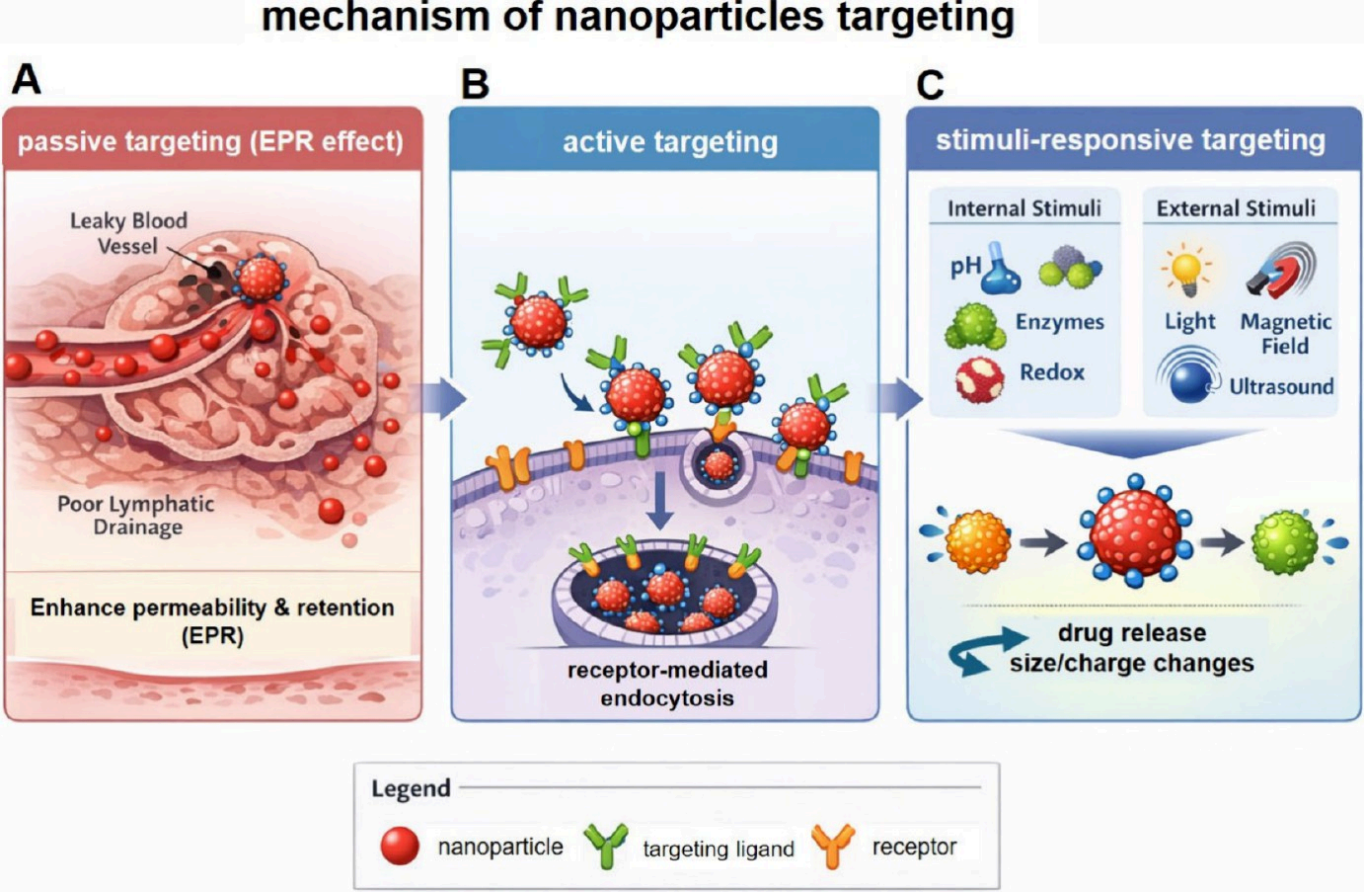
Mechanisms of nanoparticle targeting. Schematic illustration of nanoparticle targeting strategies, including passive targeting via the enhanced permeability and retention (EPR) effect, active targeting through ligand–receptor interactions on target cells, and stimuli-responsive targeting triggered by internal (e.g., pH, enzymes) or external (e.g., light, magnetic field) cues, enabling site-specific accumulation and controlled drug release. Images were generated by artificial intelligence (AI) using chat GPT.

### 5.3 Multimodal Cancer Therapy Using Nanoparticles

NPs can be synthesized in such a way that they can provide compartmentalized structures. This is beneficial for cancer therapy as cancer is a multifaceted disease, and a multimodal therapy can propose more effective treatment. Synergistic combination of several therapy approaches can overcome the limitations of monomodal therapy. One of the most common combinations that interest researchers is the combination of PTT with other therapy approaches. PTT provides significant advantages in cancer therapy for its precise spatiotemporal control and its minimal invasiveness.^204^ Plasmonic NPs such as gold (Au) and silver (Ag) NPs are the remarkable advancements for PTT method that lead to a positive clinical translation trend.^205^ To optimize the treatment, PTT is then developed to be combined with other promising methods including chemotherapy and gene therapy, in which the photosensitizer is mediating the photochemical internalization of drugs and/or gene transduction. This combination is also reported to give an enhanced cytoplasmic delivery of macromolecules through the cell membrane.^206–208^ Significant studies of multimodal cancer therapy using NPs are also depicted in comparison with the NPs-based monomodal approaches in Figure 8 as detailed references.

**Figure 8:**
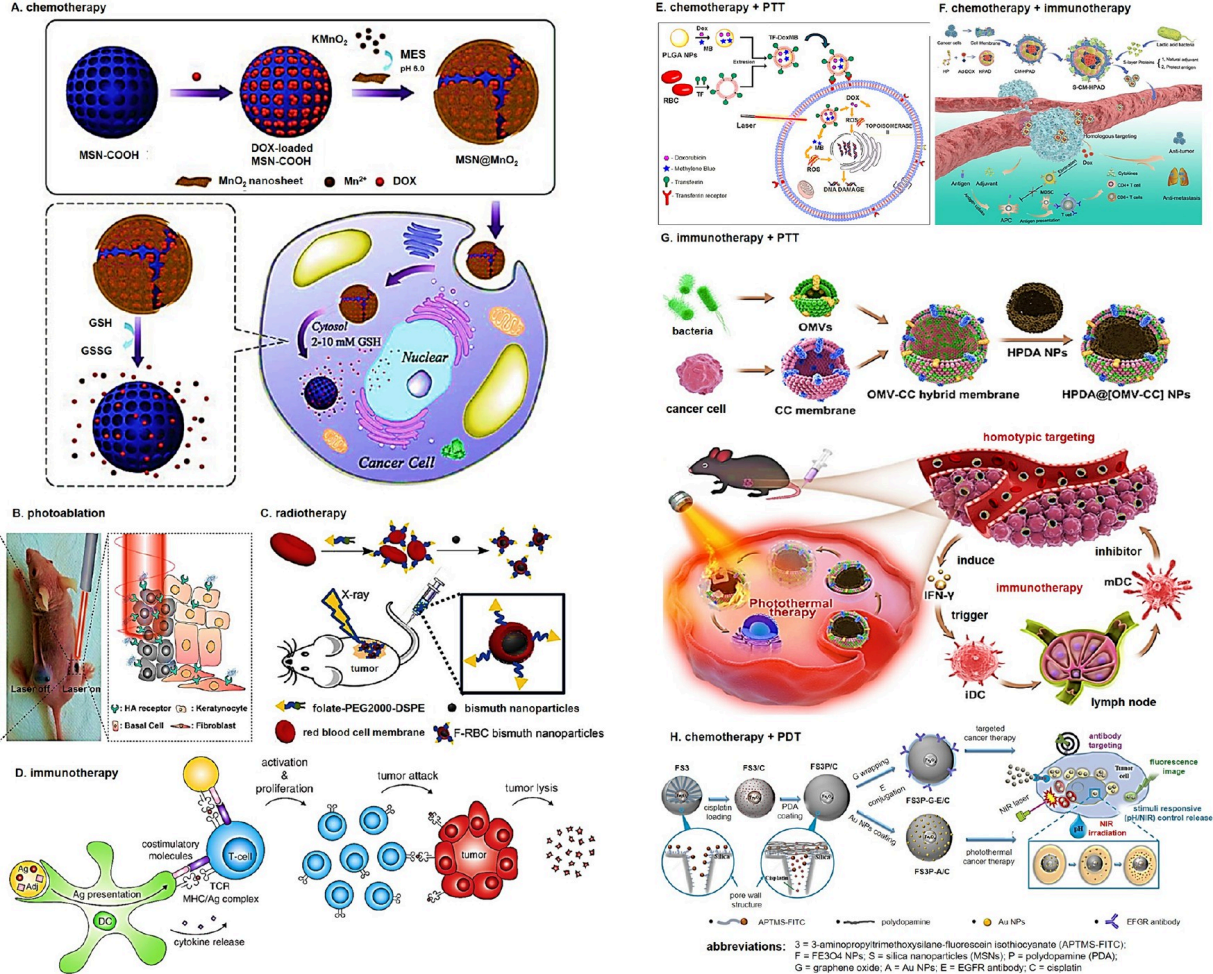
Monomodal approaches of cancer therapy using nanoparticles: (A) Mesoporous silica NPs loaded with doxorubicin as the anticancer drug and then coated with MnO_2_ which can be released when triggered by GSH inside the target cell (Adapted with permission from ref 209. Copyright 2014 John Wiley and Sons. Minor font changes from original). (B) Photothermal ablation therapy for melanoma skin cancer cell with the aid of nanographene oxide-hyaluronic conjugates (Reproduceded from ref 210. Copyright 2014 American Chemical Society). (C) Bismuth NPs modified with red blood cell membrane to enhance X-ray radiation therapy (Adapted with permission from ref 211. Copyright 2018 Elsevier./Minor font changes from original). (D) Nanovaccines were designed to deliver tumor antigens and adjuvants to the dendritic cell to stimulate T cells, or the nanovaccines can also directly modify T cells, leading to the activation of tumor-specific T cells which can destroy the tumor cells (Reproduced with permission from refs 169. Copyright 2013 Elsevier). Multimodal approaches of cancer therapy using nanoparticles: (E) Chemo and photodynamic therapy for HeLa and MCF-7 cells using transferrin conjugated red blood cell membrane-coated PLGA NPs (Reproduced from ref 212. Copyright 2020 American Chemical Society) (F) Biomimetic NPs made from doxorubicin-loaded cationic polymer NPs coated with cancer cell membrane (B16F10) and self-assembled surface layer protein from *Lactobacillus helveticus* were used to deliver both anticancer drug and tumor-specific antigen and adjuvants (Reproduced from ref 213. Copyright 2019 American Chemical Society) (G) Hollow polydopamine NPs coated with hybrid cell membrane derived from *E. coli* and B16–F10 cell underwent synergistic immune/photothermal therapy for melanoma (Adapted from ref 214. Copyright 2020 American Chemical Society. More font changes from original). (H). Core–shell of Fe_3_O_4_ and mesoporous silica NPs with double coating of polydopamine and Au were used to specifically deliver the dual stimuli-responsive drug release and photothermal therapy (Reproduced from ref 207. Available under a CC-BY-NC 4.0 license. Copyright 2020 Tran et al. More font changes from original).

## 6 Nanoparticles Used in Cancer Targeting and Therapy

There is a huge range of NPs that have been applied for cancer targeting and therapy, depending on the emphasis of the physiological properties preferred for the case. In general, there are naturally formed organic NPs such as lipids, proteins, lipoproteins, ferritins, and viruses.^215^ There are also synthetic organic NPs such as aptamers, emulsions, liposomes, solid-lipid NPs, protein-like dendrimers, nanosomes and various polymers.^215^ For inorganic NPs, there are metal-based NPs, including gold, silver, and magnetic iron. There are also mineral-based NPs such as nanohydroxyapatite and layered bimetallic hydroxides. Other types of NPs are carbon or silica nanomaterials and mesoporous dioxide. All of these mentioned NPs have been characterized as having high biocompatibility as well as low cytotoxicity. Figure 9 shows the compilation of prospective NPs in cancer therapy according to their categories. While Organic NPs provide higher biocompatibility and natural synergies with the human body, Inorganic NPs attract researchers more due to their flexibilities. Inorganic NPs are not only easy to synthesize and have acceptable biocompatibility, but also have a range of possibilities of surface functionalization, make it possible to modify them into a therapeutic agent with high drug-loading capability and controlled drug release rate. Table 8 contains numerous lists of NPs and their prospective combination, therapy methods, and target cells that have been studied in the recent development of the field.

**Figure 9:**
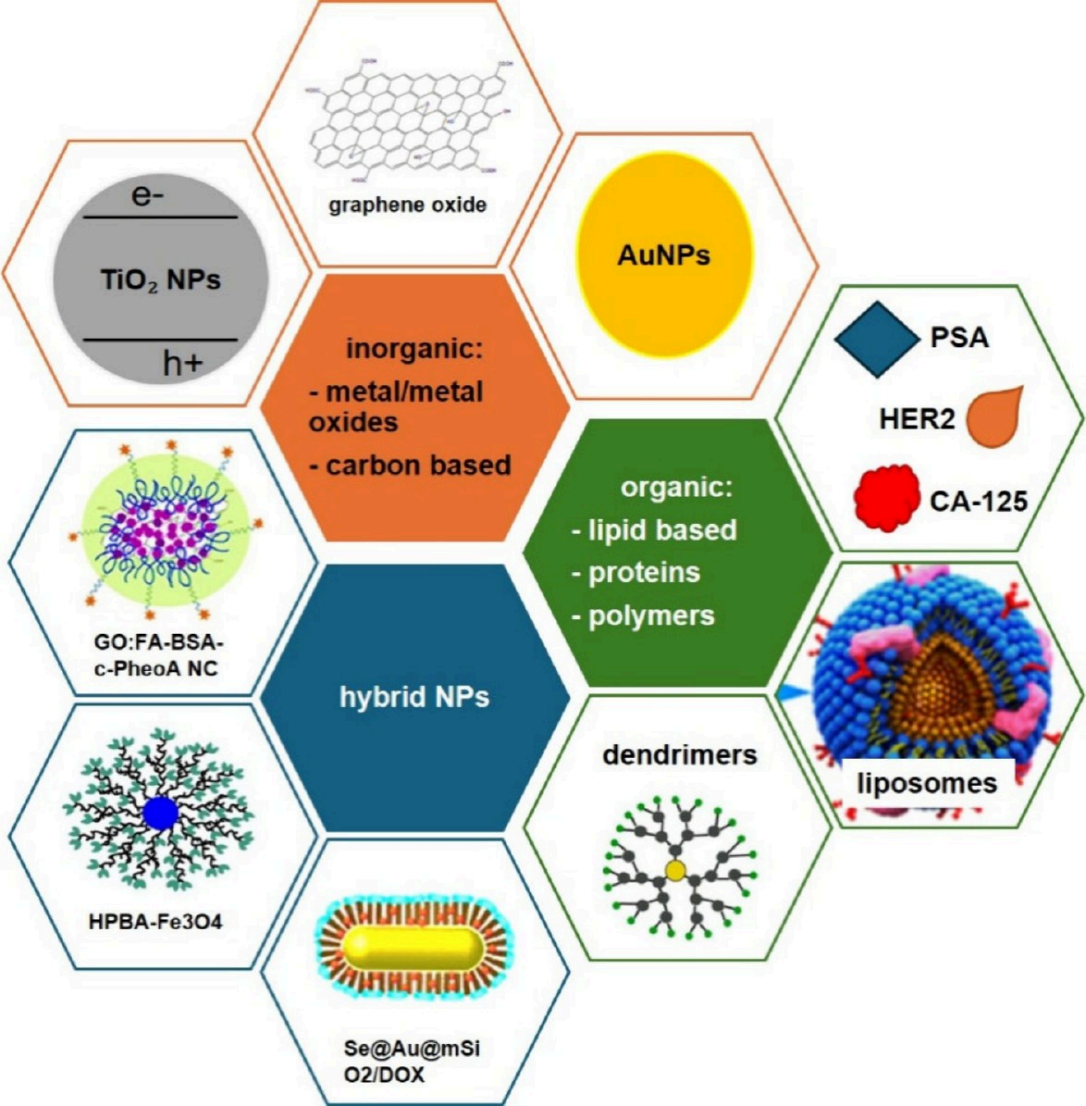
Compilation of various NPs used in cancer therapy based on the categories. Organic NPs (green cluster) include (from top right to bottom left): biomarker proteins, liposomes (Reproduced from ref 202. Copyright 2016 American Chemical Society), and dendrimers. Inorganic NPs (orange cluster) include the following (from left to right): TiO_2_ nanosemiconductors, graphene oxide (Reproduced with permission from ref 216. Copyright 2016 Elsevier), and Gold NPs. As a result of NPs tailoring, there are hybrid NPs that combines organic and inorganic NPs to enhance their targeting abilities (blue cluster). They include (from top left to bottom right): bovine serum albumin (BSA)-cis-aconityl pheophorbide-a (c-PheoA) conjugate complexed with graphene oxide (GO) to enhance antitumor efficacy as well as synergistic effect as theragnostic agents (Reproduced with permission from ref 216. Copyright 2016 Elsevier), amino-functionalized Fe_3_O_4_ magnetic nanospheres modified by hyperbranched phenylboronic acid made to enhance drug loading capacity of magnetic nanomedicines (Reproduced with permission from ref 217. Copyright 2019 Elsevier), and mesoporous silica-capped gold nanorod covered with nanoselenium overcoat NPs was fabricated as a development to incorporate materials with specific chemotherapeutic, chemoprevention, and photoablation/hyperthermia functions to enhance their synergistic effect and overcome multidrug-resistance (Reproduced from ref 206. Available under a CC BY license. Copyright 2018 Ramasamy et al.).

**Table 8:** List of Recent Nanomedicine Innovations Integrated in Various Therapy Methods^a^

No.	NPs	Particle shape/size (measurement method)	Therapy Method	Model Used	Drug Loading	Cancer Cell type	Key Outcomes	Ref
***AuNPs | Property of interest: tunable particle size, easy functionalization, surface plasmon resonance, photothermal, fluorescence, higher electron density, superparamagnetic***								
1	Pegylated silica-core gold nanoshells (pSGNs).	Diameter of 156.41 ± 15.38 nm (TEM with ImageJ software).	Hyperthermia and targeted photoablation.	In-vivo (mouse model) with external NIR laser irradiation.	–	Ovarian cancer (intraperitoneal cancers in ovarian tumor mouse models).	When conjugated with antihuman CD47 monoclonal antibody, efficacy achieved with lower pSGNs counts and shorter NIR radiation time.	^241^
2	Selenium nanoshell-capped Au@mSiO_2_ (Se@Au@mSiO_2_/DOX)	100 nm (hydrodynamic diameter/DLS).	NIR-responsive chemo-photothermal.	In-vivo (tumor-bearing female mice) with mild NIR irradiation.	Doxorubicin (DOX).	Metastatic breast cancer (MDA-MB-231)	Significant synergistic therapy effects with no noticeable adverse effect.	^206^
3	Gold–gold sulfide nanoparticles (GGS-NPs).	Bare: 42.2 nm, anti-HER2 conjugated: 63.4 nm (hydrodynamic diameter/DLS).	Targeted imaging and PTT.	In-vitro with pulsed NIR laser exposure.	–	When conjugated with anti-HER2 antibodies, specifically binds SK-BR-3 breast carcinoma which overexpressing the HER2 receptors and induced cell death.	Photoluminesence visualization of cancerous cells, followed with subsequent thermal induction on the pinpoint sites by increasing laser power from 1 mW to 50 mW, leading to precise cell apoptosis.	^312^
4	Curcumin loaded- Folic Acid- functionalized Au-PVP.	Bare AuNPs = 36.85 nm, AuPVP = 62.5 nm, CurAu-PVP = 72.56 nm (hydrodynamic diameter/DLS).	Chemotherapy.	In-vivo using preclinical breast cancer orthotopic mouse model.	Curcumin 40 μg/mL.	Breast Cancer (MCF-7 and MDA-MB-231).	Effective tumor-specific therapy without harming healthy cells.	^314^
5	Hydrphobic and positively charged AuNPs loaded with paclitaxel.	6 nm by TEM and 30–100 nm by DLS/hydrodynamic diameter, indicating different aggregation degrees.	Chemotherapy.	In-vitro.	Paclitaxel.	A549 and HeLa cells.	The hydrophobic nanoparticles trigger oxidative stress by disrupting NADPH oxidase, whereas positively charged nanoparticles do so by altering mitochondrial pathways, showing the intricate specificity of cellular stress-response mechanisms to distinct nanoparticle cues.	^315^
6	Positively charged Au–PEI-PEG-AA (Anisamide targeted).	∼110 nm (SEM).	SiRNA delivery and Chemotherapy.	In vitro using human PC3 cells and In vivo using PC3 xenograft mouse model.	Paclitaxel.	Prostate Cancer (PC3).	A coordinated suppression of the target gene, when paired with chemotherapeutic agents, produced a synergistic therapeutic benefit without introducing notable toxicity.	^316^
7	Glutathione (GSH)-coated Folic Acid-modified AuNPs loaded with methotrexate (MTX).	Spherical AuNPs= 5.6 nm MTX/Au-GSH-FA = 11 nm (hydrodinamic diameter/DLS)	Chemotherapy.	In vitro.	Methotrexate (MTX).	Human Brain (U-87) and Cervical (HeLa) cancer cells.	The synthesized nanoparticle complex demonstrates significant efficacy increase by 3 fold for U-87 MG cells and 10-fold for HeLa cells, indicating it as a promising nanocarrier agent for targeted therapy in Folate-receptor positive cancer cells.	^114^
8	AuNPs with cisplatin.	15 nm (TEM)	Chemotherapy	In vivo using xenograft mice models	Cisplatin.	Colorectal cancer (SW620 cells).	AuNPs were proven to reduce cancer-associated fibroblast density and the production of tumor stromal collagen I which leads to improved vascular perfusion and drug delivery to the tumor sites in colorectal cancel models.	^240^
***AgNPs | Property of interest: cytotoxic, tunable particle size, easy functionalization, induce oxidative stress, endocytosis, antibacterial, fluorescence, higher electron density, surface plasmon resonance***								
9	Teucrium polium leaf extract-AgNPs	70–100 nm (FE-SEM).	Chemotherapy (cytotoxicity test).	In vitro through MTT assay.	–	Gastric Cancer (MNK45)	Green alternative of AgNPs biosynthesize for medicinal and therapeutic applications.	^251^
10	AgNPs functionalized with PEG and aptamer As1411.	AgNPs= 18.82 ± 2.10 nm by TEM and 69.08 ± 0.57 nm by hydrodinamic size/DLS. AsNPs= 37.8 ± 3.13 nm (hydrodinamic size/DLS).	Radiotherapy.	In vitro using human PC3 cells and In vivo using PC3 xenograft mouse model.	–	Glioma cells (C6 glioma cells).	AsNPs were not only confirmed to only target C6 glioma cells, but also capable of penetrating into the core of the tumor, resulting in higher apoptotic cell death to the tumor but not the normal endothelial cells.	^121^
11	Mesoporous silica NPs decorated with Transferrin as nanoplatform for AgNPs immobilization (MSNs-Tf-AgNPs).	AgNPs= 15 nm (TEM), MSNs-Tf-AgNPs= 196 nm (hydrodinamic size/DLS).	Targeted chemotherapy.	In vitro.	–	Liver cancer (HepG2) with ligand: transferrin.	The nanosystem enables selective delivery of AgNPs to cancer cells with elevated transferrin receptor expression. Subsequently, the molecular mechanisms underlying the chemotherapeutic activity of the MSNs–Tf–AgNP nanocarriers were successfully elucidated.	^250^
12	AgNPs+Camptothecin (CPT)	AgNPs= 20 nm (TEM).	Combined chemotherapy.	In vitro.	Camptothecin (CPT).	Cervical cancer (HeLa).	CPT and AgNPs induce cell death through disruption of mitochondrial membrane permeability and subsequent activation of caspases 9, 6, and 3. The observed synergistic cytotoxic and pro-apoptotic effects are closely linked to elevated reactive oxygen species generation and depletion of cellular antioxidant defenses.	^247^
***Graphene Oxide (GO) | Property of interest: surface functionalization, conductivity, photothermal, fluorescence quenching, sp2 hybridization, high surface area, fast electron transfer***								
13	Nanographene-oxide -hyaluronic acid conjugate (NGO-HA).	250 nm (TEM).	Photothermal ablation.	In vitro and In vivo.	–	Melanoma skin cancer.	Complete ablation of the tumor tissues was achieved through NIR irradiation with no reoccurence.	^210^
14	Folate-bovine serum albumin (BSA)-cis-aconityl pheophorbide-a (c-PheoA)-GO conjugate (Pheo-A+GO:FA-BSA-c-PheoA NC).	182.0 ± 33.2 nm (hydrodinamic size/DLS).	Photodynamic therapy.	In vitro phototoxicity with in vivo and ex vivo bioimaging.	–	B16F10 melanoma cells and Human breast cancer (MCF-7) cells.	Folate-targeted nanoparticles exhibited markedly enhanced phototoxicity compared with free PheoA and nontargeted GO:BSA-c-PheoA nanocomposites. Consistently, PheoA + GO:FA-BSA-c-PheoA nanocomposites showed substantially greater antitumor efficacy than free PheoA or FA-BSA-c-PheoA nanoparticles under 671 nm laser irradiation.	^216^
15	HNPa loaded into Graphene Oxide(GO) coupled with magnetic Fe3O4 nanoparticles and chitosan (CS) (MCGO–HNPa).	261 ± 4.52 nm (hydrodinamic size/DLS).	Photodynamic therapy.	In vitro drug release and phototoxicity.	–	Human liver cancer cell line (HepG2 cells).	Under identical experimental conditions, the MCGO–HNPa composite exhibited a higher singlet oxygen quantum yield and enhanced cytotoxicity toward human hepatocellular carcinoma cells (HepG-2) compared with free HNPa. Cellular uptake studies further indicated that MCGO nanoparticles facilitate and accelerate the transport of HNPa into tumor cell nuclei.	^131^
16	β- cyclodextrin/cystamine/pegylated functionalized graphene oxide (GO-CysCD-PEG) with doxorubicin.	GO-CysCD= 531 nm, GO-CysCD-PEG= 564 nm (hydrodinamic size/DLS). TEM shows sheet-like structures.	Chemotherapy.	In vitro cytotoxicity.	Doxorubicin (DOX).	Human liver cancer cell line (HepG2 cells).	Cellular uptake and nuclear penetration of DOX are shown with responsive behavior toward pH and energy saturation of the cellular internalization pathways.	^317^
17	Amino acids-functionalized GO foams with cisplatin.	SEM shows foam-like structure with flake-like graphene sheets with pore size of 80 nm in average.	Chemotherapy.	In vitro drug release and MTT cytotoxicity studies.	Cisplatin.	MCF-7 and HepG2 cell lines.	Both drug loading amount and drug releasing rate are significantly increased with sustainable drug release up to 48 h.	^318^
18	Mesoporous silica nanoparticles- drug-polydopamine- graphene oxide- epidermal growth factor receptor (MSNs-drug@AF@ PDA@GO@EGFRab) with cisplatin.	MSNs@A-F@PDA@GO (without drugs)= 108 ± 4 nm by SEM, 107 ± 1 nm by DLS.	Photothemal-chemotherapy.	In vitro release test and cell viability test.	Cisplatin.	Human epithelial neuroblastoma cells (SH-SY5Y).	Stimuli-responsive controlled drug release was achieved together with higher specificity and simultaneous fluoresence imaging.	^313^
19	Superparamagnetic graphene oxide (SPMGO)- cyanuric chloride (CC) loaded with Methotrexate (MTX) (SPMGO/CC/MTX).	Magnetic nanoparticles of 9.3 ± 2.7 nm were grafted on GO nanosheets (TEM).	Chemotherapy.	In vitro drug release and MTT test.	Methotrexate (MTX).	HeLa, MCF-7, and Caov-4 cancer cell lines.	The engineered drug nanocarrier increased the efficacy of MTX chemotherapy with hemo-compatibility feature.	^319^
20	Methotrexate (MTX) loaded dopamine conjugated nano graphene oxide (DA-nGO).	nGO= 255.5 ± 56.2 nm, MTX loaded DA-nGO= 342.7 ± 14.4 nm (Photon Correlation Spectroscopy)	Chemotherapy.	In vitro drug release and cytotoxicity studies.	Methotrexate (MTX).	Human breast adenocarcinoma cell line (MCF7 cells) and Human Embryonic Kidney 293 (HEK293) cells.	Improved drug delivery efficacy for dopamine receptor positive cancer cell lines.	^320^
21	Paclitaxel (PAC) loaded Aptamer (APT)- conjugated magnetic graphene oxide (MGO) nanocarrier.	5–15 nm of magnetic nanoparticles loaded on GO sheets.	Chemotherapy.	In vitro drug release and cellular toxicity.	Paclitaxel.	MCF-7 cancer cells.	Successful specific binding of the nanocarriers with MCF-7 cancer cells compared to fibroblast normal cell.	^321^
22	Paclitaxel (PTX) loaded Pectin(PEC)- conjugated magnetic graphene oxide (PTX/PEC-GO-Fe3O4).	6–12 nm of magnetic nanoparticles loaded on GO sheets, which thickens after PEC conjugation.	Chemotherapy.	In vitro drug release and cellular toxicity.	Paclitaxel.	MCF-7 cancer lines.	The nanohybrid revealed enhanced drug release under the acidic endosomal conditions characteristic of cancer cells compared with normal physiological pH. The synthesized nanohybrid is also maintaining high levels of relative cell viability.	^322^
***Fe_3_O_4_ | Property of interest: magnetism, magnetic heat absorption, surface functionalization***								
23	Amino-functionalized Fe_3_O_4_ magnetic nanospheres modified by hyperbranched phenylboronic acid (HPBA-Fe_3_O_4_) to deliver doxorubicin.	NH_2_–Fe_3_O_4_= 40 nm with additional ∼ 4 nm thickness after hyperbranch modification (TEM).	Chemotherapy.	In vitro targeted cellular uptake.	Doxorubicin (DOX).	Glioma (U-87 MG).	Significant drug loading enhancement with simultaneuos pH responsive release and specificity.	^217^
24	VEGF-targeted nanocarrier based on albumin coated magnetic (iron oxide) nanoparticles(MNP), loaded with doxorubicin.	MNP= 8 ± 3 nm by TEM, 50 nm by AFM, 45 ± 10 nm by DLS. Discrepancies due to protein shell deformation during drying.	Theranostic chemotherapy.	In vivo using mice bearing 4T1 tumors.	Doxorubicin (DOX).	Breast adenocarcinoma (4T1).	Simultaneous cancer therapy and diagnostic utilizing hand magnetic resonance imaging (MRI).	^265^
25	Chitosan-coated magnetic nanoparticles (CCMNPs) loaded with black pomegranate peel extract.	Uncoated MNPs= 30 ± 7.1 nm, after chitosan coating (CCMNPs)= 34.2 ± 8.4 nm, the BPPE loaded CCMNPs= 37.3 ± 6 nm (DLS).	Chemotherapy.	In vitro drug loading and release, MTT and LDH assays.	Black Pomegranate Peel Extract (BPPE).	Various breast cancers (MBA-MB-231, NIH/3T3, 4T1 cells).	The developed drug-loaded magnetic nanoparticles show significant efficacy to eradicate cancer cells compared to free drug delivery, with both cases show no toxic effect to the normal cells.	^266^
26	Metformin/Gemcitabine-Fe_3_O_4_-pH low inserton peptide.	PEGylated MNPs = 18.9 nm, after drug loading= 22.9 nm (DLS).	Chemotherapy.	In vitro cell studies and in vivo using orthotopic PDAC tomur-bearing nude mice.	Gemcitabine (GEM).	Pancreatic cancer (PANC-1).	The two-step delivery strategy specifically for pancreatic cancers has been developed and proven to be effective with the use of Metformin to enhance the penetration of Gemcitabine into the target sites.	^267^
***ZnO | Property of interest: cytotoxicity, reactive oxygen species, photothermal, surface functionalization, biocompatible***								
27	Lipid-coated ZnO.	Bare ZnO = 14 ± 2 nm by FESEM and 55 nm by DLS, after lipid-coating = 21 ± 5 nm by FESEM and 110 nm by DLS.	ROS-generator in Photodynamic therapy.	In vitro cellular uptake.	–	Human epithelial carcinoma (HeLa cells).	Lipid coating enhances colloidal stability of ZnO NPs. The lipid coated ZnO NPs successfully internalized in target cells and effectvely kill them under UV activation.	^275^
28	Rubia tinctrorum synthesized ZnO.	10 to 70 nm, average 40 nm	induce apoptosis			breast cancer (MDA-MB-231)		
***Mesoporous Silica (MSN) | Property of interest: porous, easy functionalization, heat absorption, biodegradable***								
29	Mesoporous silica NPs (MSN) encapsulation.	Bare MSN= 61.85 ± 5.89 nm by TEM, 61 nm by DLS. Phosphonate modified MSN= 72 nm (DLS) and amine modified MSN= 267 nm due to aggregation (DLS).	Chemotherapy.	In vitro release, antiproliferative and citotoxicity.	Docetaxel (Doc).	Prostate cancer (PC3).	Negatively charged phosphate modified MSNs show superior control over drug release with enhanced antiproliferative activity. The combination of polyphenols (RES) and anticancer drugs (Doc) can be achieved using MSNs to improve cancer therapy efficacy on resistant cancers.	^284^
30	Curcumin-loaded mesoporous silica nanoparticles (MSNPs)	PEG-MSNPs-Cur= 184.6 ± 13.69 nm (DLS).	Chemotherapy.	In vitro studies on HepG2 and HeLa cells and in vivo studies on untreated and treated tumor-bearing mice.	Curcumin (Cur).	HepG2 and HeLa cells.	MSNPs improve curcumin bioavailability while protecting it from premature degradation. The findings demonstrate that curcumin-loaded nanoparticles significantly enhance tumor growth inhibition.	^283^
31	Chondroitin sulfate (ChS)-coated MSN	MSNs-ChS= 224.6 ± 0.7 nm, MSNs-ChS@PQ= 227.2 ± 2.9 nm (DLS).	Chemotherapy.	In vitro studies, in vivo and ex vivo drug distribution and in vivo chemotherapy and safety study.	Paclitaxel combined with Quercetin.	MCF-7/ADR cells	Multidrug Resistancy of the tumor cells was effectively reversed through the combination of paclitaxel and quercetin which inhibits P-gp and thus increase drug penetration and retention in tumor cells.	^186^
***Copper | Property of interest: cytotoxicity, higher electron density, photothermal, functionalization***								
32	PLGA coated folate-mediated multiple drug loaded CuO nanoparticles.	CuO NPs= 115.24 2.3 nm, FOL-PLGA-CuO NPs= 164.98 1.3 nm, drug loaded NPs ranges from 180 to 195 nm depending on the PLGA concentration (TEM).	Chemotherapy.	In vitro.	Docetaxel and Doxorubicin.	Nasopharangeal cancer (CNE-2).	PLGA coating and folic acid conjugation have increased the overall NPs specific targeting, with the concentration og PLGA involved govern the drug release.	^323^
33	Copper diethyldithiocarbamate (Cu(DDC)2) complex NPs.	40–80 nm depending on drug loading and stabilizer concentration (DLS).	Chemotherapy.	In vitro.	Disulfiram.	Prostate cancer (DU145-TXR), MCF-7 breast cancer, MDA-MB-231 breast cancer cells.	A SMILE (stabilized metal ion ligand complex) method was developed for the fabrication of Cu(DDC)2 nanoparticles, which exhibit strong anticancer potential for drug-resistant cancers via paraptosis. The SMILE technology is aimed to help simplified NPs preparation to ease the clinical translation.	^324^
34	Cu-based MOF, with carbon defect concentration with optimum photothermal efficiency at 800 °C (Cu@CPP-800).	Cu NPs = 90 ± 38 nm (TEM), Cu@CPP-800 = 260 ± 10 nm by TEM, 290 ± 10 nm by DLS).	Photothermal Therapy with NIR irradiation.	In vivo using tumor bearing nude Balb/c mice.	–	HeLa cells.	Remarkable photothermal conversion efficiency for carbon-based phothermal agents, with positive results on NIR driven phototherapy for cancer treatment.	^282^
***CMNPs | Property of interest: high targeting ability, controllable drug release***								
35	Indocyanine green (ICG)-loaded and cancer cell membrane-coated nanoparticles, (ICNPs).	Spherical core–shell structure with hydrodynamic size of 200.4 nm (DLS).	Photothermal therapy and diagnostic (theranostic) under NIR.	In vitro and in vivo.	–	MCF-7 cells.	The cancer cell membrane reduced interception of kidney and liver while enhance tumor site accumulation by homologous cell adhesion. Simultaneous imaging and photothermal ablation were achieved with deep cell penetration and high spatial resolution.	^200^
36	Surface-layer (S-layer) prepared from lactobacillus strains protein-enhanced immunotherapy strategy based on cell membrane-coated (S-CM-HPAD nps).	S-CM-HAPD polymers ∼200 nm (TEM, SEM, AFM, CLSM), the S-layer has monolayer thickness of 4–5 nm (TEM).	Chemo-immunotherapy.	In vitro and in vivo with C57BL/6 mice.	Doxorubicin (DOX).	Murine B16F10 melanoma, human A549 lung cancer, rat C6 glioma, human gastric cancer SGC 7901, and human Hep G2 liver cancer cell lines.	The biomimetic NPs not only perform hopmotypic targeting, but also activate multiantigenic immunity and inhibit tumor growth and metastasis.	^213^
37	Albumin nanoparticles (ANPs) coated with macrophage plasma membranes (RANPs) loaded with paclitaxel (PTX).	ANPs = 138.7 ± 3.5, and the RANPs (after membrane coating) = 188.7 ± 4.8 nm (DLS).	Chemotherapy.	In vitro and in vo using melanoma bearing female mice.	Paclitaxel (PTX).	Murine melanoma cell line B16F10.	The engineered NPs exhibit prolonged circulation time without premature drug release. Improved tumor targetability was also shown with high compatibility with various drug compounds and varying physiochemical properties. Macrophage membrane coatings significantly increased tumor cells uptake.	^325^
38	Fused membrane materials derived from red blood cells (RBCs) and melanoma cells (B16–F10 cells) camouflaged doxorubicin (DOX)-loaded hollow copper sulfide nanoparticles (DCuS@[RBC–B16] NPs).	Bare CuS NPs = 190 nm, CuS@[RBC-B16] NPs = 200 nm, the thickness of the outer membrane shell = ± 8 nm (all measured by TEM).	Synergistic photothermal/chemotherapy.	In vitro and in vivo using nude mice.	Doxorubicin (DOX).	B16–F10 cells (melanoma).	Specific self-recognition to the target cells (B16–F10 cells) were achieved with prolonged circulation life. The synergystic therapy approach demonstrated a high efficacy up to 100% tumor growth inhibition rate.	^208^
39	Macrophage-Membrane-Coated Emtansine Liposome (MEL)	Hydrodynamic diameter of the emtansine liposome = 64.5 nm, and for MEL = 115.4 nm.	Chemotherapy.	In vitro and in vivo using lung metastatic breast cancer model mice.	Emtansine.	Metastatic 4T1 breast cancer cells.	The enginnered nanoparticles have successfully perform improved specific targeting of the metastatic foci in the lung and markedly suppressed the metastasis tumor growth.	^202^
40	Transferrin-conjugated red blood cell membrane-coated PLGA NPs with doxorubicin (Dox) and methylene blue (MB) loading (TF-DoxMB NPs).	Hydrodynamic size of PLGA NPs = 104 nm, average size of spherical TF-DoxMB NPs = 106 ± 23 nm (DLS).	Chemo-photodynamic therapy.	In vitro.	Doxorubicin (DOX).	HeLa and MCF-7 cells.	Transferrin conjugation over red blood cell membrane exhibited high stability and specificity as the tumor targeting agent. The laser irradiation contribute in the production of ROS leading to DNA damage of the cells and thus increase the therapy outcome.	^212^
41	Red blood cell membrane-modified bismuth (F-RBC bismuth NPs).	Bismuth NPs = 40 nm (TEM), F-RBC bismuth NPs = 56 nm (DLS).	X-ray radiation therapy.	In vitro and in vivo using tumor-bearing female mice.	–	Breast cancer (4T1 cells).	F-RBC bismuth nanoparticles induced greater cancer cell damage under X-ray irradiation by promoting enhanced ROS generation and exhibited prolonged blood circulation with increased tumor accumulation. Their use as X-ray sensitizers resulted in significant tumor growth inhibition and improved survival rates in mice. It was demonstrated that the nanoparticles were effectively cleared from the body within 15 days, with no observable tissue damage or inflammatory responses in major organs.	^211^
42	Outer membrane vesicle (OMV) derived from *E. coli* DH5α and Cancer cell (CC) membrane from B16–F10 cell fused to obtain an OMV-CC hybrid membrane and encapsulate it onto hollow polydopamine (HPDA) NPs (HPDA@[OMV-CC] NPs).	HPDA NPs = 180 nm (TEM), with increased hydrodynamic size of 20 nm after coating (DLS).	Synergistic immuno-photothermal therapy.	In vitro and in vivo using melanoma bearing mice.	–	A549 cells, MCF-7 cells and B16–F10 cancer cells.	Leveraged specific targeting with simultaneous immune activation via rapidly stimulating dendritic cell maturation. Combined with photothermal therapy, complete melanoma eradication was achieved using the developed NPs.	^214^
43	Semiconducting polymer (poly(benzobisthiadiazole-*alt*-thiophene)) nanoengager coated with pre-engineered 4T1 tumor cell and dendritic cells (SPNE), first phototherapeutic nanoparticles with multicellular engagement ability.	Before membrane coatings = 54.2 nm, after membrane coatings = 75.2 nm (DLS).	Synergistic immuno-photothermal (NIR-II) therapy.	In vitro immunce cell priming and tumor cell ablation followed by in vivo photothermal immunotherapy on tumor-bearing mice.	–	4T1 tumor cells.	SPNE was directly cited on the lymph nodes and target tumors to exert dual vaccination effects. With photoirradiation, SPNE further improves immune development againt distant tumors.	^143^
***TiO_2_ | Property of interest: reactive oxygen species, oxidative, porous, heat absorption, easy functionalization***								
44	Ag-doped TiO_2_ NPs.	Pure TiO_2_ = 15 nm, Ag-doped TiO_2_ = 9 nm (FETEM). DLS measurement shows 10–15 times larger particle size than FETEM due to agglomeration.	Chemotherapy.	In vitro.	–	Human liver cancer (HepG2), human lung (A549) and breast (MCF-7) cancer cells.	Ag-doped TiO_2_ exhibited selective toxicity toward cancer cells while sparing normal cells. The toxicity in human liver cancer cells was induced via oxidative stress.	^326^
45	TiO2-NPs, 86% anatase/14% rutile.	24 ± 6 nm	Chemotherapy (long exposure, low concentration).			lung alveolar epithelial cells.		^327^
46	Zinc phthalocyanine- integrated TiO2 nanoparticles (ZnPc/ZnPc-TiO2).	∼100 nm (SEM).	Photodynamic therapy.	In vitro.	–	Mouse mammary carcinoma (EMT6), human cervical adenocarcinoma (HeLa).	The synthesized nanoparticles showed profound success in their role as both photodynamic therapy agent and nuclear imaging agent.	^273^
47	Titanium dioxide nanoparticles (TiO_2_ NPs) in a layer of folic acid (FA).	TiO2 NPs = 161 nm, FA-TiO2 NPs = 37 nm (DLS).	Chemotherapy.	In vitro.	–	T24 cells (bladder cancer).	Folic acid coating on TiO2 not only enables targeting but also enhance cytotoxicity and apoptosis %.	^263^
***Quantum Dots | Property of interest: unique photophysical characteristics, ultrasmall size, enhanced permeability and retention (EPR) effect, high surface flexibility, strong and tunable fluorescence, and lower toxicity profile***								
48	CuInSe2@ZnS:Mn quantum dots (QDs).	4.6 ± 0.6 nm (TEM)	Photothermal + photodynamic therapy.	In vitro and in vivo using tumor bearing mice.	-	4T1 breast cancer tumors.	The quantum dot nanoplatform showed successful simultaneous tumor visualization and eradication with the ability to induce antitumor immune response to prevent recurrence.	^328^
49	Hybrid QD@MSN-EPI-Au-PEG-Apt nanocarriers.	65.67 ± 3.70 nm (DLS).	Radiotherapy + chemotherapy.	In vitro strudies, in vivo using immunocompromised HT-29 tumor-bearing mice and ex vivo fluorecense imaging.	Epirubicin (EPI).	Human colorectal cancer cells (HT-29).	On top of the successful selective drug delivery and active targeting radiosensitizers, the hybrid nanoparticles were equipped with pH-controlled release mechanism and potential mechanism of degradation to completely be removed from the body. Their excellent traceability via Magnetic Resonance, Computed Tomography, and Fluoresence imaging modalities were also studied.	^329^
***Carbon Nanotubes | Property of interest: electrical, mechanical, and optical properties, smaller size and defined structure, larger surface area and tubular morphology, and unique photothermal conversion efficiency***								
50	Doxorubicin-loaded PEGylated-multiwalled-carbon nanotube (DOX-PEG-MWCNTs-3).	Fiber-like nanotube with length of ∼ 300 nm (TEM) and hydrodinamic diameters of 273 nm (DLS). PEGylated process adds slight thickness to the tube width.	Chemotherapy.	In vitro.	Doxorubicin (DOX).	HepG2 cells.	Targeted and pH-triggered drug release were demonstrated by the developed nanoparticles with increased inhibitory efficiency against the target cancer cells.	^294^
51	Acid-functionalized multiwalled-carbon nanotubes (ox-MWCNTs).	Net-forming tubulars with width <100 nm (FESEM).	Hyperthermia.	In vitro histological analysis and in vivo using female Balb.c miceinoculated with EMT6 cancer cells.	–	EMT6 murine breast tumor.	The ox-MWCNTs were demonstrated as an effective local hyperthermic agent to eradicate EMT6 breast tumor which also promote immunce cells infiltration within the tumor microenvironment.	^295^
52	Single walled nanotube coupled with Cyanine7 and IGF-1R antibody (SWNT-CY7-IGF-1Ra).	Fiber-like with width <100 nm (TEM).	Imaging-guided photothermal therapy.	In vitro and in vivo using orthotopic pancreatic cancer(BXPC-3)-bearing mice.	–	Pancreatic cancer (ASPC-1, BXPC-3 and PANC-1).	The developed SWNT hybrid can spesifically accumulate on tumor sites and completely spare the normal cells. The aggregations inside the tumor cells allow for dynamic locating of the tumor, leading to accurate and efficient laser irradiation.	^296^

a DLS = Dynamic Light Scattering, TEM = Topography Electron Microscopy, SEM = Scanning Electron Microscopy, FE SEM = Field Emission Scanning Electron Microscopy, AFM = Atomic Force Microscopy, CLSM = Confocal Laser Scanning Microscopy), MTT = 3-(4,5-dimethylthiazol-2-yl)-2,5-diphenyltetrazolium bromide, LDH = lactate dehydrogenase, NIR = Near Infrared.

### 6.1 Organic Nanoparticles

Organic nanoparticles offer several advantages for cancer diagnosis and therapy, setting them apart from nucleic-acid–based treatments and inorganic NPs. Their small size, sustained effect, high payload capacity, and surface functionalization potential enable precise drug delivery and targeting.^218,219^ As selective carriers and energy converters—particularly in photodynamic therapy (PDT)^220^—organic NPs enhance cancer cell death and therapeutic outcomes. To address challenges such as poor water solubility and drug biocompatibility, therapeutic agents can be encapsulated within polymeric or lipid-based NPs. Especially for active targeting functionalization, organic NPs are the main candidates due to the incorporated moieties that naturally interact with specific receptors within the target cells. Promising organic NP platforms include polymeric micelles, block copolymer micelles, liposomes, and biologically derived nanoproteins, all of which show potential in improving targeted cancer therapies.

#### 6.1.1 Lipid-Based Nanoparticles

Liposomes are extensively employed lipid-based nanoparticles, forming circular vesicles with one or more bilayer membranes composed mainly of natural or synthetic phospholipids.^221^ These vesicles have amphiphilic characteristics, and their surfaces can be easily altered to enhance drug delivery procedures. Liposomes have become preferred vehicles for chemotherapeutic agents, and currently, several liposomal drugs are in clinical use. One such example is emtansine, an anticancer drug, which was encapsulated in pH-responsive liposomes and coated with macrophage membranes.^222,223^ This innovative approach resulted in improved targeting abilities and effectively suppressed lung metastasis.^202^ Incorporating particular liposomes into nanoscale particles improved the uptake of these particles by cancer cells. In contrast, solid lipid nanoparticles have restricted solubility in aqueous environments, requiring surfactants for practical usage.^224^ Solid lipid NPs are composed of triglycerides, lipids, fatty acids, steroids, and waxes, and they can be programmatically released at specific time intervals.^225^ These NPs can be delivered to targeted anatomical sites through various methods including ingestion, injection, or other suitable routes.

#### 6.1.2 Protein-Based Nanoparticles

Proteins are complex organic biomolecules made up of intricate peptide chains and possess intricate four-dimensional structures. Proteins play a crucial role in cancer targeting and therapy due to their biological functions, specificity, and interactions with cellular and molecular targets. Their biological origin gives them remarkable biocompatibility, biological activity, low immunogenicity, and significant potential for drug loading and stability.^226^ These inherent features make proteins highly beneficial for use as NPs in cancer therapy and targeting. Protein NPs have been widely utilized, including albumin, ferritin, whey protein, lipoproteins, silk protein, peptides and other varieties.^227^

Proteins offer essential insights into the presence, type, and stage of cancer, enabling them to serve as biomarkers. For example, elevated levels of CA-125 indicate ovarian cancer, Prostate-Specific Antigen (PSA) is linked to prostate cancer, and HER2/neu overexpression is associated with certain breast cancers.^228–230^ Proteins expressed by the cells are highly related to the cell mechanism and metabolism; this explains why different proteins are found in different types of cells. By utilizing the appropriate proteins, one can easily target specific cells due to the specific interaction that the proteins have with the target cells.

Proteomics advancements further refine the analysis of protein expression in cancer, leading to new biomarker discoveries and improved cell targeting precision. Protein-based cancer therapy supports personalized medicine by tailoring strategies to construct individual protein profiles, followed by designing a personalized treatment strategy. However, challenges remain in achieving high specificity for cancer cells and efficient methods to modify the proteins to be applicable in clinical treatment, such as scalability, reproducibility, and regulatory approval.

#### 6.1.3 Polymer-Based Nanoparticles

Polymer-based nanoparticles, made from either biodegradable or nonbiodegradable materials, often consist of an oily or aqueous core enclosed by a polymeric shell. Their advantages lie in tunable features, such as size, surface characteristics, and photosensitizer release rates, which can be tailored through material selection. Therapeutic agents can be incorporated into these NPs by covalent attachment, encapsulation, or postloading methods.^231^ Depending on their properties, drugs are loaded into the core or shell via hydrophobic interactions, electrostatic forces, or hydrogen bonding, especially in the case of hydrophobic agents, which rely on compatibility with the polymer matrix. Additionally, the integration of glycopolymeric structures like glycodendrimers and glycoclusters has shown promise in photodynamic therapy for breast cancer by enhancing NP binding affinity to carbohydrate receptors on cancer cells.^220^

Polymer-assisted NPs play a crucial role as drug carriers in various biomedical applications. For their successful application, these NPs must demonstrate biocompatibility and nontoxicity, while also possessing suitable physical structures. The choice of polymers is vital, taking into account their half-lives to ensure no leakage of impurities and promoting biodegradability. Apart from the challenges, polymer-assisted NPs offer several advantages, including exceptional stability and the ability for large-scale production. In cancer therapeutics, polymer-assisted NPs find diverse applications, including core–shell polymeric NPs, dendrimers, polymersomes, polyplexes, and nanogels.^222^ For example, the biodegradable polymer poly­(lactic glycolic acid) (PLGA) is frequently used in NPs synthesis. It is produced by the polycondensation of lactic acid and glycolic acid in different proportions. PLGA has received approval from the US Food and Drug Administration (FDA) and is widely employed in clinical medicine for various applications.^232^

### 6.2 Inorganic Nanoparticles

Inorganic nanoparticles (INPs) offer distinct advantages in cancer therapy by enhancing drug delivery through targeted ligands while reducing off-target effects via controlled adsorption and infiltration.^233^ Initially developed as drug carriers, INPs have evolved into multifunctional platforms with intrinsic anticancer properties, including cytotoxicity, oncogene suppression, and inhibition of cancer-related signaling pathways.^234^ Their ability to codeliver a wide range of therapeutic agents—such as chemotherapeutics, nucleic acids (siRNA, miRNA, shRNA), and photosensitizers—without interference or degradation makes them highly versatile.^233^ Functionalization with ligands, polymers, and biomolecules further improves their targeting and therapeutic efficiency. Additionally, their optical and magnetic properties support applications in molecular imaging and externally guided delivery. INPs also exhibit inherent gene-silencing and tumor-inhibitory effects, positioning them as promising tools in the development of synergistic cancer treatments that integrate therapies and diagnostics.

#### 6.2.1 Metal-Based Nanoparticles

In cancer therapy, the ability to tailor treatment to individual tumor profiles is increasingly important. Metal nanoparticles (MNPs) have emerged as a versatile platform in this context, offering precise control over shape, size, charge, and surface chemistry.^235^ Such tunability enhances the targeting efficiency and therapeutic performance. MNPs also possess unique optical properties driven by surface plasmon resonance, leading to strong absorption across the visible to the near-infrared (NIR) spectrum. This makes them highly attractive for biomedical applications such as imaging, diagnosis, and therapy. Their magnetic and photosensitive nature allows for use in cancer imaging, photothermal therapy, and photodynamic therapy.^236^ MNPs, including gold and silver, also offer high surface area, which supports drug loading, enrichment, separation, and targeted delivery.^237^ Additionally, their magnetothermal properties enable indirect destruction of cancer cells, offering further promise for therapeutic applications. Functionalizing MNPs with a specific ligand further improves their selective accumulation in tumor tissues, supporting more effective drug delivery and reducing off-target effects. Notably, MNPs show higher cellular uptake than similarly sized nonmetallic nanoparticles, which enhances their efficiency as drug carriers.^155,238^ This facilitates not only targeted therapy but also enables simultaneous diagnosis and treatment thanks to their strong optical signals and drug-release capabilities. These features position MNPs as powerful tools in the development of next-generation cancer nanomedicine.

There is a growing interest in nanoparticles made of noble metals, with a strong focus on gold nanoparticles (AuNPs).^239^ These nanoparticles exhibit versatile properties and hold great potential for applications in clinical chemistry, bioimaging, cancer therapy, and targeted drug delivery and are continuously being investigated and characterized. Gold, being a noble metal, is known for its low reactivity compared to those of other chemical elements. The synthesis of AuNPs has been highly refined, enabling precise control over their size, shape, and dimensions. This allows researchers to customize AuNPs with specific properties suited to different targets in cancer cells. The significance of using AuNPs with various sizes and shapes for cancer therapy applications is continuously increasing and has shown remarkable potential in both drug delivery and simultaneous diagnosis. Extensive evidence supports the notion that AuNPs can effectively prevent drug degradation, improve drug solubility, prolong circulation time in the bloodstream, and enable controlled release of drugs.^157^ The enhancement of cisplatin delivery to a colorectal cancer model has been enabled with AuNPs as reported by Zhao et al.^240^ Their study proved that AuNPs help to reduce solid stress within tumors, thereby improving blood vessel perfusion. The modification of the shape of gold nanoparticles also results in diverse optical properties across the visible to near-infrared (NIR) spectrum, making them valuable tools for applications in bioimaging, thermognostic, and monitoring the morphological properties of AuNPs during synthesis. Ramasamy et al. has demonstrated that AuNPs could effectively accumulate in tumors, with the release of the therapeutic payload controllable remotely via mild NIR irradiation when tailored as triple-combination nanosized Se\@Au\@mSiO2/DOX particles.^206^ Moreover, Wu et al.^241^ demonstrated that conjugating pegylated silica-core gold nanoshells pSGNs with an antihuman CD47 monoclonal antibody significantly enhances the photoablative effect, enabling tumor ablation with a reduced dose of pSGNs and shorter NIR laser exposure. Repeated photothermal treatment has shown effective elimination of intraperitoneal tumors in mouse models of ovarian cancer without harming normal tissues. Another intriguing effect associated with the shape of gold nanoparticles, influenced by surface plasmon bands, is the modulation of fluorescence properties by nearby fluorophores.^242,243^ This can be attributed to various phenomena, including fluorescence resonance energy transfer (FRET), photoinduced electron transfer (PET) processes, and the photothermal properties arising from light absorption and subsequent nonradiative energy dissipation.^244^ This unique surface-enhanced optical properties and the ability to interact with nearby fluorophores open up exciting possibilities for advancing various cancer treatment applications of AuNPs.

Silver nanoparticles (AgNPs) have also gained attention for their unique chemical and physical characteristics, particularly in biomedical applications. They are generally nontoxic to human body and possess strong antibacterial activity.^245^ Recent studies have highlighted their potential in cancer treatment, where AgNPs can enter cancer cells via endocytosis and exert cytotoxic effects through mechanisms such as oxidative stress induction, DNA damage, mitochondrial disruption, and apoptosis.^121,246–251^ In a notable study, Gholami et al.^252^ demonstrated the slow release of pseudomonas exotoxin (PE38) from AgNPs over 100 h, resulting in dose-dependent cytotoxicity and apoptosis in MCF-7 cells. AgNPs synthesized using *Sesbania grandiflora* leaf extract caused immediate cellular damage in MCF-7 cells, including oxidative stress, membrane disruption, and cell death. Similarly, AgNPs loaded with berberine showed cytotoxic effects on MCF-7 and MDA-MB-231 cells, with in vivo tests confirming tumor suppression and minimal toxicity to normal tissues.^253^ Another study investigated nanofibers incorporating niclosamide and AgNPs (nic@Ag NPs), which showed enhanced anticancer activity compared to separate administration.^254^ Further research explored quercetin-loaded, folate-targeted AgNPs (QRC-FA-AgNPs) for breast cancer therapy, revealing efficient delivery to MDA-MB-231 cells and significant tumor inhibition compared to free quercetin.^255^ Despite these encouraging outcomes, more studies are needed to clarify their therapeutic mechanisms and assess their long-term safety for clinical use.

#### 6.2.2 Metal Oxides Nanoparticles

Other than the stable noble metals, metal oxide nanoparticles also have emerged as innovative therapeutic anticancer agents, either alone or in combination with other compounds. Metal oxide nanoparticles, including titanium dioxide, iron oxide, zinc oxide, copper oxide, and silicon dioxide present a promising approach by improving targeted drug delivery, minimizing systemic toxicity, and alleviating chemotherapy-related side effects such as neurotoxicity and cardiotoxicity.^256^ When conjugated with drugs, these nanocarriers can enhance drug delivery efficiency and prolong circulation time in the bloodstream.^256^ Their beneficial physiochemical properties enable precise targeting, efficient drug delivery, and enhanced imaging, leading to better treatment outcomes with reduced side effects and drug resistance. Advances in metal oxide synthesis and functionalization have improved their stability and drug-loading capacity, making them effective tools in cancer therapy.

Titanium dioxide nanoparticles (TiO_2_ NPs), typically around 100 nm in size, are known for their strong photocatalytic properties when activated by UV light, making them useful in fields like medicine, energy, and biosensing.^257^ In cancer therapy, they have gained attention for their ability to target tumor cells in acidic environments and release anticancer drugs inside cells through redox mechanisms. TiO_2_ NPs can also generate reactive oxygen species (ROS), which damage cancer cells by attacking proteins, lipids, and DNA, contributing to oxidative stress. However, their reliance on UV light for activation limits their effectiveness due to poor tissue penetration and potential health risks. To address these issues, researchers are working on modifying TiO_2_ NPs to absorb visible or near-infrared (NIR) light, which can penetrate deeper into tissues far from the skin surface and extend their cancer-fighting effects.^258–260^ Strategies include combining TiO_2_ with graphene oxide (GO) or doping it with metals like tungsten, nickel, or gold to reduce the energy needed for activation and improve ROS generation.^261^ These modifications also help prolong the ROS lifespan and enhance the therapeutic impact. Preventing nanoparticle aggregation using stabilizers is also important, as clumping can reduce the surface area and lower the effectiveness. For better drug delivery, TiO_2_ NPs can be functionalized with anticancer drugs like paclitaxel or doxorubicin,^262^ and modified with targeting molecules like folic acid^263^ to improve selectivity and uptake by cancer cells that overexpress folate receptors. The findings revealed that efficient targeting of FA–TiO2 NPs enhanced cellular internalization, leading to increased apoptosis in T24 cells (bladder cancer).^263^

Iron oxide nanoparticles are widely used in parenteral iron therapy, especially alongside erythropoietin-stimulating agents,^264^ to help manage anemia in patients with end-stage renal disease and cancer. Clinical studies suggest that using these nanoparticles in cancer patients may not only improve anemia but also boost survival rates, possibly through more than just correcting low hemoglobin. Some research points to the idea that these nanoparticles could help activate the immune system, particularly cells like macrophages, which might play a role in fighting tumors.^265^ This effect is similar to how the body responds to mild infections and could help the immune system recognize and attack cancer cells. Iron oxide nanoparticles are already approved for medical use and are valued for their safety, ability to break down naturally in the body, and their use in imaging, like MRI, thanks to their magnetic properties.^217,265–267^ This magnetic property is especially useful to target tumors in challenging locations such as brain tumors, as proven by previous studies.^268,269^ However, a major challenge is that they can carry only a small amount of drug, which limits how effective they are unless large (and potentially harmful) doses are used. Some newer systems, like hyperbranched PBA-based designs, have improved drug loading, targeting, and controlled release, helping to address this issue.^217^ Targeting strategies using molecules like anti-VEGF antibodies have also shown promise, increasing how well drugs reach tumors and improving results in animal studies.^265^ Moreover, Taherian et al. have shown the highly improved drug delivery efficacy using magnetic nanoparticles, which enables delivery control and nontoxic toward normal cells.^266^

Zinc plays a vital role in DNA repair, oxidative stress regulation, and apoptosis through proteins like p53 and caspases.^270^ Its deficiency is linked to increased cancer risk, while zinc oxide nanoparticles (ZnO NPs) offer a promising approach for cancer therapy due to their ability to selectively induce oxidative stress in cancer cells.^271^ ZnO NPs generate reactive oxygen species (ROS) and release zinc ions intracellularly, disrupting protein function and triggering cell death. These effects are amplified in cancer cells due to their higher baseline ROS and negative surface charge.^272–274^ Their biocompatibility, ease of synthesis, and potential for surface modification make them suitable for photodynamic therapy (PDT) and drug delivery, although UV activation still limits their tissue penetration. Recent studies have explored innovative designs to enhance the therapeutic performance. One study developed lipid-coated ZnO NPs, which improved stability in physiological media and efficiently entered HeLa cells.^275^ Upon UV activation, they generated ROS and killed cancer cells at nontoxic doses. Another study synthesized porous rod-shaped ZnO NPs using palm fruit extract.^276^ These green-synthesized particles showed anticancer activity against HT-29 and MCF-7 cells, with in vivo tests confirming their safety when loaded with doxorubicin.

Copper oxide nanoparticles (CuO NPs) have attracted an increasing interest in cancer therapy due to their ability to induce selective cytotoxicity in malignant cells.^277^ Their high surface-area-to-volume ratio and redox activity enable the generation of, like all the previous oxides, reactive oxygen species (ROS), mitochondrial dysfunction, and activation of apoptotic pathways such as p53 and Bax/Bcl-2, leading to effective tumor cell death.^278^ Studies have demonstrated their antitumor effects across various cancer types, including breast, liver, pancreatic, and lung cancers.^279^ Notably, CuO NPs have shown the ability to target tumor-initiating cells (TICs), disrupt the cell cycle, and suppress tumor growth in vivo. Beyond engineered CuO NPs, the role of copper (Cu) itself in cancer biology is notable. Cu is an essential trace element involved in redox enzymes and mitochondrial respiration. However, elevated copper levels have been consistently observed in cancer patients, along with increased Cu/Zn, Cu/Se, and Cu/Fe ratios, suggesting a link between Cu homeostasis and tumor progression.^280^ In one study, Copper nanoparticles (CuNPs) were found to degrade DNA through a singlet oxygen–mediated mechanism even in the absence of external agents, emphasizing their potential as redox-active.^281^ Engineered composites such as Cu@CPP-800—composed of partially oxidized CuNPs supported on porous carbon—have demonstrated efficient cellular uptake, high photothermal conversion under NIR light (48.5%), and effective tumor ablation in animal models without significant dark toxicity. These systems also offer dual mode imaging capabilities, showing potential as multifunctional platforms in theranostics.^282^

Silica nanoparticles (SiNPs) consist of silicon dioxide, found in widespread applications in various fields including medical theragnostic and drug delivery.^154^ Their advantages include large surface area, controllable particle size, and excellent biocompatibility. Biomedical applications of SiNPs have been extensively studied, focusing on surface and structural modifications to target specific cancers and enable disease diagnosis and therapy.^154,186,283,284^ Silica nanoparticles are considered as effective carriers due to their high biocompatibility and ability to carry a large drug load, due to their well-defined mesoporous and hollow structures. In vitro studies using the HepG2 human liver cancer cell line showed that doxorubicin-loaded in mesoporous silica with MnO_2_ capsulation effectively entered the cells and released the drug, resulting in higher cytotoxicity compared to normal human liver cells (L02).^209^ However, it should be noted that silica nanoparticles can potentially be toxic to the rest of the body; hence, their toxicity still needs to be extensively evaluated.

#### 6.2.3 Carbon-Based Nanoparticles: Graphene

Graphene-based nanoparticles, specifically graphene oxide nanoparticles (GO NPs), have displayed significant potential in the field of chemotherapy. These nanoparticles exhibit distinctive optical and physicochemical characteristics, including an amplified surface area, biocompatibility, modifiable active groups, and a robust photothermal effect.^285^ As a result, they can act as active components and versatile carriers for advanced drug delivery and cancer therapy.^286^ The characteristics of GO NPs make them highly suitable for improving treatment effectiveness and facilitating targeted drug delivery in cancer therapy. Enzalutamide-loaded graphene oxide quantum dots (QDs) were formulated, resulting in improved drug-loading efficiency and growth inhibition in prostate cancer cell lines C4–2B and LNCaP.^287^ Furthermore, these nanoparticles displayed an enhanced ability to target tumors and decreased side effects of enzalutamide in in vivo experiments.^287^ Similarly, curcumin-loaded graphene nanoparticles, especially in the structure of graphene quantum dots, demonstrated a synergistic effect in inducing cell death of Kerman male breast cancer and MCF-7 tumor cells, surpassing the impact of raw curcumin utilized in the therapy.^288^ These studies demonstrate the potential of graphene-based nanoparticles as effective tools for improving drug delivery and enhancing therapeutic outcomes in cancer treatment.

#### 6.2.4 Carbon-Based Nanoparticles: Carbon Nanotube

Since their discovery by Sumio Iijima in 1991, carbon nanotubes (CNTs) have emerged as powerful nanomaterials with wide-ranging applications in electronics, materials science, and biomedicine.^289^ Structurally, CNTs are cylindrical allotropes of carbon formed by rolling graphene sheets into seamless tubes. They exist as either single-walled carbon nanotubes (SWCNTs) or multiwalled carbon nanotubes (MWCNTs), depending on the number of concentric graphene layers. Their diameters typically range from 0.4 to 100 nm, with lengths extending to several micrometers.^290^

Due to their exceptional electrical, mechanical, and optical properties, CNTs have attracted increasing attention in the field of oncology for drug delivery, photothermal therapy, gene delivery, and diagnostic imaging.^291^ SWCNTs, owing to their smaller size and defined structure, are particularly useful for drug and gene delivery, while MWCNTs, with their larger surface area and tubular morphology, can be employed for higher payload loading and photothermal conversion.^292^

However, native CNTs are hydrophobic and prone to aggregation in biological fluids, which limits their bioavailability and raises toxicity concerns. To overcome these challenges, both covalent and noncovalent functionalization strategies have been employed to improve their solubility, biocompatibility, and circulation time.^293^ PEGylation—functionalization with polyethylene glycol—is a particularly effective method to reduce immunogenicity, extend biological half-life, and enhance passive tumor accumulation via the enhanced permeability and retention (EPR) effect.^294^

PEGylated CNTs have shown remarkable efficacy in cancer models. For instance, Zhao et al. formulated PEGylated MWCNTs for the intracellular tumor-targeted release of doxorubicin (DOX). While pristine MWCNTs exhibited cytotoxicity, PEGylated variants (≤300 nm) demonstrated enhanced cytocompatibility, high drug-loading capacity, and improved anticancer activity against HepG2 cells.^294^ Similarly, acid-functionalized MWCNTs combined with localized hyperthermia achieved complete tumor regression in a murine breast cancer model, with elevated Hsp70 expression and enhanced immune responses at the tumor site.^295^

Functionalized CNTs can also be tailored for targeted photothermal therapy. In one study, SWCNTs were conjugated with Cy7 fluorophore and IGF-1R antibody (SWNT-CY7-IGF1-Ra) for near-infrared (NIR) imaging-guided therapy of pancreatic cancer.^296^ Upon intravenous injection, these nanoprobes exhibited sustained tumor retention and strong fluorescence signals up to 48 h postadministration, outperforming nontargeted controls in tumor accumulation and biodistribution.^296^ Metabolism studies revealed liver and intestinal processing of the conjugates, while normal tissues showed minimal uptake, confirming selective tumor localization.^296^

Beyond chemotherapy and imaging, CNTs are gaining traction in use in gene delivery and immunotherapy. Functionalized CNTs have demonstrated the ability to cross cellular and even blood-brain barriers, delivering cargos such as plasmid DNA, siRNA, antisense oligonucleotides, and aptamers to specific cellular targets.^297^ A notable example includes the delivery of cyclosporin A (CsA) using amine-terminated phospholipid–PEG chains conjugated to SWCNTs via a cleavable ester bond, offering controlled drug release and reduced systemic toxicity.^298^

The unique photothermal conversion efficiency of CNTs in the NIR region enables their use in thermal ablation therapy. Upon NIR laser irradiation, CNTs efficiently absorb energy and convert it into heat, inducing localized tumor cell death with minimal damage to surrounding tissues.^299^ This has made CNTs ideal candidates for PTT, either alone or in combination with chemotherapeutic agents, to maximize therapeutic efficacy.^299^

In diagnostics, CNTs show promise as biosensors and contrast agents. Their inherent optical and electronic properties make them excellent candidates for early cancer detection and intraoperative imaging. Functionalized CNTs have been used to detect cancer biomarkers and map tumor vasculature, contributing to improved diagnostic precision.^300^

#### 6.2.5 Quantum Dot

Quantum dots (QDs) are semiconductor nanocrystals composed of hundreds to thousands of atoms, typically derived from group II–VI elements such as CdSe or ZnS.^301^ Structurally, they consist of a core made of semiconductor material, a shell (often ZnS) to enhance optical properties and stability, and an outer capping layer that can be functionalized with targeting ligands or polymers such as polyethylene glycol (PEG).^301^ These nanostructures exhibit unique photophysical characteristics, including high quantum yield, broad absorption spectra, narrow emission peaks, and exceptional photostability, making them ideal candidates for applications in biomedical imaging and drug delivery.^302,303^

Their ultrasmall size (2–10 nm) allows QDs to passively accumulate in tumor tissues via the enhanced permeability and retention (EPR) effect. This characteristic also makes them effective tracers in drug delivery systems. Their surface can be flexibly modified with a wide array of targeting ligands, such as peptides, folic acid, and monoclonal antibodies, enabling site-specific delivery to tumors.^303^ Furthermore, the strong and tunable fluorescence of QDs facilitates real-time monitoring of drug release and biodistribution, significantly advancing the prospects of image-guided therapy. Despite rapid advancements, one of the main limitations remains the lack of standardized, scalable, and reproducible synthesis methods, especially for biocompatible QDs.

Functionalized QDs are increasingly being explored in multimodal applications, including fluorescence imaging, photodynamic therapy, biosensing, and targeted drug and gene delivery. In bioimaging, QDs outperform traditional dyes due to their superior brightness, longer fluorescence lifetimes, and resistance to photobleaching. For example, manganese-doped CuInSe/ZnS QDs functionalized with folic acid have demonstrated near-infrared II (NIR-II) fluorescence with efficiencies up to 31.2% and simultaneous high contrast in magnetic resonance imaging (MRI), underscoring their potential as multifunctional theranostic agents.^304^

QDs have been effectively utilized in imaging diverse cancer types, including breast, ovarian, glioblastoma, pancreatic, and hepatocellular carcinomas. Anti-IGF1R-conjugated QDs have shown promise in targeting and visualizing breast cancer cells. Similarly, QDs targeting EGFR or HER2—receptors commonly overexpressed in epithelial tumors—have enabled the fluorescent imaging of malignant cells and in vivo studies of tumor pharmacokinetics.^305^

Among various types of QDs, carbon-based quantum dots—such as graphene quantum dots, nanodiamond QDs, and carbon quantum dots—are gaining attention due to their favorable biocompatibility and rapid excretion. Graphene quantum dots, in particular, are widely used in cancer research owing to their ease of functionalization and lower toxicity profile. One prominent example includes an aptamer-doxorubicin-quantum dot conjugate engineered for targeted prostate cancer therapy.^306^

Photodynamic therapy using QD-based delivery systems offers an additional therapeutic route by enabling precise control over drug release kinetics, thereby reducing the frequency and intensity of side effects. Nanoscale QDs have also been explored for use in nasal and systemic delivery systems that enhance the bioavailability and tumor targeting. Innovations such as nanoparticle perfluorocarbon (nanoPFC)-based delivery systems are showing enhanced success rates in treating a variety of tumors, achieving therapeutic efficacy between 68–80% depending on tumor type and delivery route.^307^

Furthermore, QDs can serve as nanoprobes for NIR bioimaging and image-guided surgery, with the capacity for multimodal applications, including angiogenesis imaging and tumor vascular mapping. Notably, QD-assisted early tumor detection methods have demonstrated diagnostic accuracies exceeding 90% when combining optical density with fluorescence imaging.^308^

### 6.3 Hybrid Nanoparticles

Hybrid nanoparticles (NPs) are structured nanomaterials engineered by integrating two or more distinct nanocomponents, each offering specific physicochemical or biological functionalities. This modular architecture allows hybrid NPs to perform multiple therapeutic roles simultaneously—such as drug delivery, photothermal therapy, gene silencing, or imaging—within a single platform.^309^ Such versatility is increasingly harnessed in nanomedicine to implement multimodal or combination therapies, which are designed to overcome the limitations of monotherapies, enhance tumor specificity, and reduce off-target toxicity. Current research focuses on developing new types of hybrid NPs that are precisely designed to maximize drug loading in tumor cells.^310,311^ This aligns with the main idea of targeted delivery optimization, that is, to reduce side effects on healthy cells, improving overall therapeutic outcomes. By integration of the features of multiple nanocomponents, hybrid NPs present a promising approach for more effective and selective drug delivery in cancer treatment and other medical applications. In most studies, if not all, the use of NPs is developed in a hybridization manner to achieve higher effectivity as different NPs compensate each other, as tabulated in Table 8.

Recent innovations in hybrid NP systems have demonstrated impressive potential in cancer treatment by coupling therapeutic modalities (Figure 10). For example, Se@Au@mSiO_2_/DOX nanoparticles integrate selenium-capped gold cores within a mesoporous silica matrix and are loaded with doxorubicin to enable chemo-photothermal therapy responsive to near-infrared (NIR) irradiation. This construct not only enhances drug accumulation in metastatic breast cancer (MDA-MB-231) but also provides localized thermal ablation for synergistic tumor eradication.^206^ Similarly, gold–gold sulfide nanoparticles (GGS-NPs) conjugated with anti-HER2 antibodies deliver targeted imaging and photothermal therapy (PTT) for HER2-overexpressing breast carcinoma (SK-BR-3), combining real-time diagnostics with treatment precision.^312^

**Figure 10:**
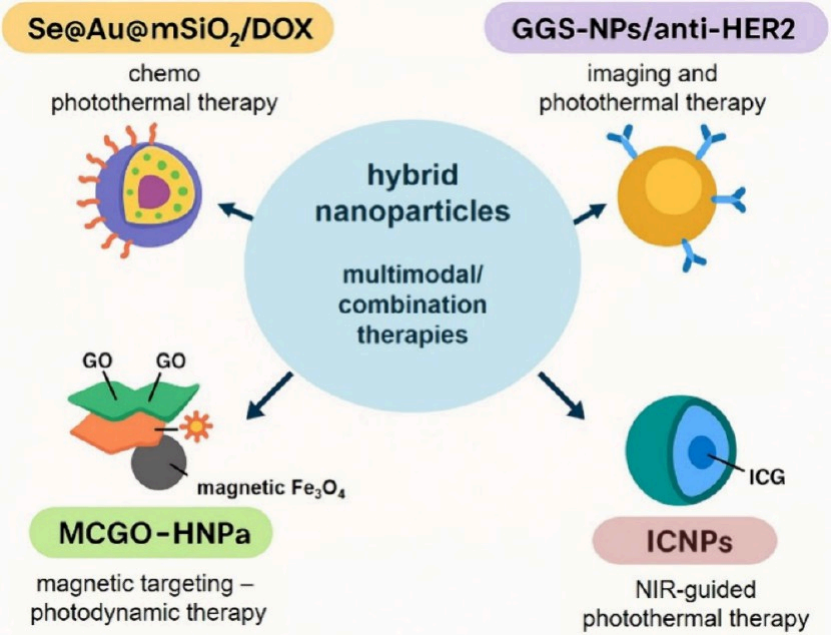
Illustration depicting different types of hybrid nanoparticles tailored to enhance the cancer therapy efficacy. These include combinations such as metallic–polymeric, lipid–polymeric, and inorganic–organic hybrids, each designed to integrate the complementary properties of their constituent materials. Such designs enable improved drug loading and stability, targeted delivery to tumor sites, controlled and stimulus-responsive drug release, and the potential for multimodal therapeutic actions (e.g., chemotherapy combined with photothermal or photodynamic therapy). Some examples depicted include selenium-capped gold cores within a mesoporous silica matrix integrated with loads of doxorubicin for a responsive chemo-photothermal therapy (top left), gold–gold sulfide nanoparticles coupled with anti-HER2 antibodies enabling targeted imaging and photothermal therapy (top right), ICG-loaded cancer membrane-coated nanoparticles (bottom right) exploit tumor-derived membranes to target homological tumor cells, and magnetic Fe_3_O_4_, graphene oxide (GO), and photosensitizers were integrated to enable simultaneous magnetic targeting and photodynamic therapy (bottom left).

Graphene-based hybrids also exemplify a multimodal capability. The MCGO–HNPa system—comprising magnetic Fe_3_O_4_, graphene oxide (GO), and photosensitizers—enables both magnetic targeting and photodynamic therapy (PDT) for HepG2 cells, taking advantage of GO’s large surface area and Fe_3_O_4_’s magnetic properties for spatial control and light-activated cytotoxicity.^131^ Moreover, The multifunctional Mesoporous silica nanoparticles system—featuring fluorescent cores, polydopamine, and graphene oxide (GO) shells—enables targeted, pH/NIR-responsive drug delivery for neuroblastoma cells, leveraging GO’s high surface area for enhanced uptake and controlled drug release, and fluorescent labeling for imaging-guided photothermal therapy.^313^

Hybrid NPs also support chemo-immunotherapy strategies, as shown by outer membrane vesicle (OMV)–cancer cell membrane-coated polydopamine nanoparticles (HPDA@[OMV-CC]), which deliver localized heat and immune stimulation under NIR exposure to treat melanoma and breast cancer simultaneously.^214^ Further, ICG-loaded cancer membrane-coated nanoparticles (ICNPs) utilize tumor-derived membranes for homologous targeting, while indocyanine green facilitates NIR-guided photothermal therapy, making them ideal for theranostic use in MCF-7 breast cancer.^200^

These and many other hybrid systems (Table 8) demonstrate that rational nanocomponent integration can achieve synergistic effects across therapy types, improving clinical relevance and reducing reliance on single-modality treatments. Such multifunctional approaches represent a strategic shift in cancer nanomedicine, prioritizing not just effectiveness but also adaptability and precision in overcoming tumor heterogeneity.

## 7 Clinical Translation of Cancer Nanomedicines: From Bench to Bedside

Despite decades of intensive research, only a limited number of nanoparticle-based therapeutics have achieved clinical translation. To bridge this gap, Table 9 summarizes representative FDA- and EMA-approved cancer nanomedicines, highlighting their formulation strategies, clinical indications, and key translational lessons.^330^ Collectively, these examples demonstrate that clinical success has been driven predominantly by improvements in pharmacokinetics, drug stability, and toxicity reduction rather than by highly specific active targeting. Liposomal formulations such as Doxil/Caelyx, Myocet, and Onivyde exemplify how nanocarriers can prolong circulation and mitigate dose-limiting toxicities, while Vyxeos underscores the clinical importance of maintaining synergistic drug ratios through coencapsulation.^330^ In contrast, ABRAXANE illustrates that carrier-free nanoparticle formulations can achieve broad clinical adoption by improving solubility and eliminating toxic excipients with efficacy largely governed by altered biodistribution. Importantly, these approved systems also reveal critical manufacturing and quality challenges that shape translational outcomes. Scalability, batch-to-batch variability, sterility assurance, endotoxin testing, and reproducibility of surface functionalization remain major barriers to widespread clinical deployment. From a regulatory perspective, nanoparticle-based therapeutics are subject to stringent chemistry, manufacturing, and control (CMC) and good manufacturing practice (GMP) requirements, necessitating well-defined physicochemical characterization, long-term stability, and tightly controlled production processes. These constraints have limited the clinical progression of many promising nanomedicine platforms.

**Table 9:** Approved Cancer Nanomedicines Highlight Formulation Strategies and Clinical Translation Challenges

Nanomedicine (Trade Name)	Nanocarrier/Formulation Type	Clinical Indication (S)	Approval Year	Regulatory Agency	Key Translational Lessons
**Doxil/Caelyx**	PEGylated liposome	Ovarian cancer, Kaposi’s sarcoma, multiple myeloma	1995 (FDA), 1996 (EMA)	FDA, EMA	Demonstrated prolonged circulation and reduced cardiotoxicity; highlighted variability of EPR-based tumor accumulation and challenges in batch-to-batch reproducibility
**ABRAXANE**	Albumin-bound nanoparticles	Breast cancer, NSCLC, pancreatic cancer	2005 (FDA)	FDA	Showed that carrier-free “nanoparticle” formulations can improve solubility and avoid toxic solvents; clinical success driven by pharmacokinetics rather than active targeting
**Onivyde**	Liposomal irinotecan	Metastatic pancreatic adenocarcinoma	2015 (FDA)	FDA	Validated nanocarrier-enabled drug repurposing; underscored importance of controlled release and dosing to overcome rapid drug clearance
**Vyxeos**	Liposomal coencapsulation (fixed drug ratio)	High-risk acute myeloid leukemia	2017 (FDA), 2018 (EMA)	FDA, EMA	Highlighted the clinical value of maintaining synergistic drug ratios in vivo; a benchmark for combination nanomedicine design
**Myocet**	Non-PEGylated liposome	Breast cancer	2000 (EMA)	EMA	Reduced cardiotoxicity compared to free doxorubicin; limited circulation time emphasized the trade-offs between PEGylation and clearance
**DaunoXome**	Liposome	AIDS-associated Kaposi’s sarcoma	1996 (FDA/EMA)	FDA, EMA	Demonstrated feasibility of liposomal anthracyclines but limited impact on long-term survival outcomes
**Hensify**	Hafnium oxide nanoparticles	Locally advanced soft tissue sarcoma	2019 (EMA)	EMA	Introduced nanoparticles as physical radioenhancers rather than drug carriers; highlighted regulatory acceptance of nondrug nanomaterials
**NanoTherm**	Iron oxide nanoparticles	Brain tumors	2011 (EMA)	EMA	Demonstrated localized, intratumoral nanotherapy; limited scalability emphasized delivery-route constraints

Taken together, the clinical landscape outlined in Table 9 suggests an implicit translation checklist for future nanomedicine development, encompassing: (i) robust and scalable manufacturing routes; (ii) reproducible surface chemistry and formulation consistency; (iii) comprehensive in vitro and in vivo safety evaluation; and (iv) early alignment with regulatory expectations. Addressing these factors at early stages of development is essential to improving the likelihood of successful clinical translation. Looking forward, emerging modalities offer new opportunities to overcome the existing translational bottlenecks. AI-driven nanoparticle design is increasingly being used to optimize formulation parameters, predict biodistribution, and reduce experimental trial-and-error. In parallel, multiomics-guided targeting strategies are enabling a more biologically informed selection of nanoparticle properties tailored to tumor heterogeneity. Furthermore, the growing synergy between nanomedicine and immunotherapy—through immune-modulating nanoparticles and nanoenabled vaccine platforms—represents a promising frontier for achieving durable antitumor responses. Integrating these emerging approaches with clinically validated design and manufacturing principles will be critical for advancing the next generation of cancer nanomedicines from bench to bedside.

## 8 Patient Stratification and Biomarker-Guided Nanomedicine Alternative

The clinical variability of tumor microenvironments necessitates patient-specific strategies to maximize nanoparticle delivery and therapeutic efficacy. Imaging and molecular diagnostics provide critical tools for stratifying patients and guiding nanomedicine selection. Functional imaging modalities such as PET (Positron Emission Tomography), MRI (Magnetic Resonance Imaging), or hybrid PET/MRI can quantify tumor perfusion, vascular permeability, and interstitial pressure, enabling the identification of tumors likely to exhibit favorable EPR-mediated accumulation. Complementary molecular profiling, including receptor expression mapping or genomic and proteomic analyses, informs the selection of ligand-targeted nanoparticles tailored to tumor-specific biomarkers, optimizing cellular uptake and subcellular trafficking. Furthermore, AI-driven patient profiling can integrate multiomic and imaging data to predict NP biodistribution, clearance, and therapeutic response, supporting personalized dosing strategies and combination therapies. Collectively, these approaches facilitate biomarker-guided nanomedicine deployment, transforming passive NP administration into a precision oncology platform that accounts for interpatient heterogeneity in tumor permeability, ECM density, and molecular target availability.

## 9 Conclusion and Outlook

This review comprehensively highlights the evolving role of nanoparticles in modern cancer therapy. By exploring the unique hallmarks of cancer biology, limitations of conventional therapies, and the wide-ranging capabilities of nanomedicines, we have outlined how NPs offer an unparalleled opportunity to overcome many of the longstanding challenges in oncology. Nanoparticles, through their physicochemical versatility and capacity for surface modification, have enabled significant progress in drug delivery, guided tumor imaging, and therapy enhancement. Their ability to passively accumulate in tumors via the EPR effect and to actively target cancer cells via ligand-mediated binding has opened new frontiers for precise, patient-specific intervention. The ability of NPs to bypass biological barriers has also enabled significant innovations in drug delivery, imaging, and multimodal treatment strategies. Integrations opportunity with chemotherapy, photothermal therapy, gene therapy, and immunotherapy demonstrates the feasibility of tailored interventions that maximize efficacy while minimizing side effects. Moreover, biomimetic and stimuli-responsive NP systems show tremendous promise in improving the selectivity and addressing tumor heterogeneity.

While preclinical progress has been impressive, translating these innovations into clinical practice remains a major challenge. Looking ahead, several key directions warrant our attention:

1. **Translational bottlenecks**: Scalable and reproducible nanoparticle synthesis, regulatory approval pathways, and cost-effective manufacturing remain critical hurdles. Streamlined fabrication processes and standardization will be essential to move promising NPs from bench to bedside.
2. **Multifunctional nanocarriers and AI-guided personalization**: Integrating multiple therapeutic modalities—chemotherapy, immunotherapy, gene therapy, and photothermal therapy—into single, responsive nanocarriers holds great potential. AI-driven design and patient-specific modeling can further optimize dosing, targeting, and therapeutic outcomes.
3. **Reproducibility and patient-specific targeting**: Ensuring batch-to-batch consistency, predictable pharmacokinetics, and robust targeting across heterogeneous tumors is vital. Advanced imaging validation and biomarker-guided monitoring will support clinical decision-making and improve safety profiles.
4. **Clinical validation and imaging integration**: The convergence of nanoparticle platforms with real-time imaging and diagnostics can provide quantitative measures of accumulation, therapeutic response, and tumor heterogeneity, accelerating regulatory approval and clinical adoption.

### 9.1 Future Research Roadmap

To realize the full potential of nanoparticles in cancer therapy, future studies should prioritize scalable and standardized production methods, multifunctional designs capable of sequential or combinatorial therapy, systematic evaluation of patient-specific variability, and integrated imaging-guided monitoring. Addressing these challenges will pave the way for truly personalized, efficient, and safe nanoparticle-based interventions, shaping the next generation of oncology treatments.
